# Head-to-head comparison of different classes of FAP radioligands designed to increase tumor residence time: monomer, dimer, albumin binders, and small molecules vs peptides

**DOI:** 10.1007/s00259-023-06272-7

**Published:** 2023-06-01

**Authors:** Jacopo Millul, Lennart Koepke, Gaonkar Raghuvir Haridas, Konstantin M. J. Sparrer, Rosalba Mansi, Melpomeni Fani

**Affiliations:** 1 Division of Radiopharmaceutical Chemistry, University Hospital Basel, Basel, Switzerland; 2 Institute of Molecular Virology, Ulm University Medical Center, Ulm, Germany

**Keywords:** Fibroblast activation protein-α (FAP), Radioligand therapy, FAPI, Dimer, Albumin binder, FAP-2286

## Abstract

**Purpose:**

Fibroblast activation protein-α (FAP)-targeting radioligands have recently demonstrated high diagnostic potential. However, their therapeutic value is impaired by the short tumor residence time. Several strategies have been tested to overcome this limitation, but a head-to-head comparison has never been done. With the aim to identify strengths and limitations of the suggested strategies, we compared the monomer FAPI-46 versus (a) its dimer (FAPI-46-F1D), (b) two albumin binders conjugates (FAPI-46-Ibu (ibuprofen) and FAPI-46-EB (Evans Blue)), and (c) cyclic peptide FAP-2286.

**Methods:**

^177^Lu-labeled ligands were evaluated in vitro in cell lines with low (HT-1080.hFAP) and high (HEK-293.hFAP) humanFAP expression. SPECT/CT imaging and biodistribution studies were conducted in HT-1080.hFAP and HEK-293.hFAP xenografts. The areas under the curve (AUC) of the tumor uptake and tumor-to-critical-organs ratios and the absorbed doses were estimated.

**Results:**

Radioligands showed IC_50_ in the picomolar range. Striking differences were observed in vivo regarding tumor uptake, residence, specificity, and total body distribution. All [^177^Lu]Lu-FAPI-46-based radioligands showed similar uptake between the two tumor models. [^177^Lu]Lu-FAP-2286 showed higher uptake in HEK-293.hFAP and the least background. The AUC of the tumor uptake and absorbed dose was higher for [^177^Lu]Lu-FAPI-46-F1D and the two albumin binder conjugates, [^177^Lu]Lu-FAPI-46-Ibu and [^177^Lu]Lu-FAPI-46-EB, in HT1080.hFAP xenografts and for [^177^Lu]Lu-FAPI-46-EB and [^177^Lu]Lu-FAP-2286 in HEK293.hFAP xenografts. The tumor-to-critical-organs AUC values and the absorbed doses were in favor of [^177^Lu]Lu-FAP-2286, but tumor-to-kidneys.

**Conclusion:**

The study indicated dimerization and cyclic peptide structures as promising strategies for prolonging tumor residence time, sparing healthy tissues. Albumin binding strategy outcome depended on the albumin binding moiety. The peptide showed advantages in terms of tumor-to-background ratios, besides tumor-to-kidneys, but its tumor uptake was FAP expression–dependent.

**Supplementary information:**

The online version contains supplementary material available at 10.1007/s00259-023-06272-7.

## Introduction

The tumor microenvironment (TME) is a complex fundamental part of solid tumors [1] whose composition is different among patients. Nevertheless, there are common phenotype analogies among individuals [2, 3]. Stromal cells and extracellular matrix are the main component of the TME, in which cellular infiltrates such as lymphocytes, macrophages, adipocytes, and fibroblasts are present [1]. Cancer-associated fibroblast (CAF) is one of the most abundant cell type in the TME, heavily contributing to the whole tumor mass [4]. CAFs are characterized by the expression of fibroblast activation protein-α (FAP), which is a type II transmembrane serine protease found in more than 90% of epithelial tumors such as breast, lung, colorectal, pancreatic, and ovarian cancer. FAP expression in healthy tissues and in non-malignant tissues surrounding the tumor is very limited, as confirmed by immunohistochemistry [5, 6]. Thus, FAP has recently been identified as a pan-tumoral agent. A class of small molecule–based radioligands targeting FAP has emerged in the last few years for imaging of solid tumors [7, 8]. The value of these radioligands has been illustrated in more than one hundred patients with unprecedented tumor-to-organ selectivity. Thus, FAP-targeting radioligands have been recently dubbed “potential novel molecule(s) of the century” [8, 9].

While their potential as imaging agents is undeniable, their potential for therapy is harmed by the short retention in the tumor, leading to suboptimal tumor radiation doses and, thus, limited efficacy [7, 13,10-]. A promising strategy to improve the tumor retention is via the increase of radioligand’s avidity for its target by dimerization of the binding moiety [14-16]. Another strategy involves the introduction of an albumin binder moiety, such as Evans Blue, which increase the exposure of the tumor to the radioligand due to its higher blood circulation [17, 18]. Alternatively, first-in-human results of the cyclic peptidic structure [^177^Lu]Lu-FAP-2286 showed high and persistent uptake in primary and metastatic tumor [19, 20].

While many preclinical and first-in-human clinical data have been generated with these radioligands, a comprehensive, comparative study to understand the strengths and limitations among the mentioned strategies has never been performed.

Here, we compared head-to-head representative FAP-targeting radioligands from each strategy that was proposed to prolong tumor residence time. More specifically, using [^177^Lu]Lu-FAPI-46 as the reference small molecule, we compared it with (a) a dimeric version of it, (b) two conjugates of it with different albumin binders, and (c) the [^177^Lu]Lu-FAP-2286, as the representative peptide-based radioligand. Head-to-head in vitro and in vivo assessments were performed using two cell lines characterized by low and high FAP expression, respectively. Our aim was to identify the strengths and limitations of the different strategies, namely, dimerization, albumin binder conjugation, and peptides vs small-molecule monomers, for the development of FAP-targeting radiotherapeutics.

## Material and methods

### Synthesis, radiolabeling, and log *D* determination

**Fig. 1:**
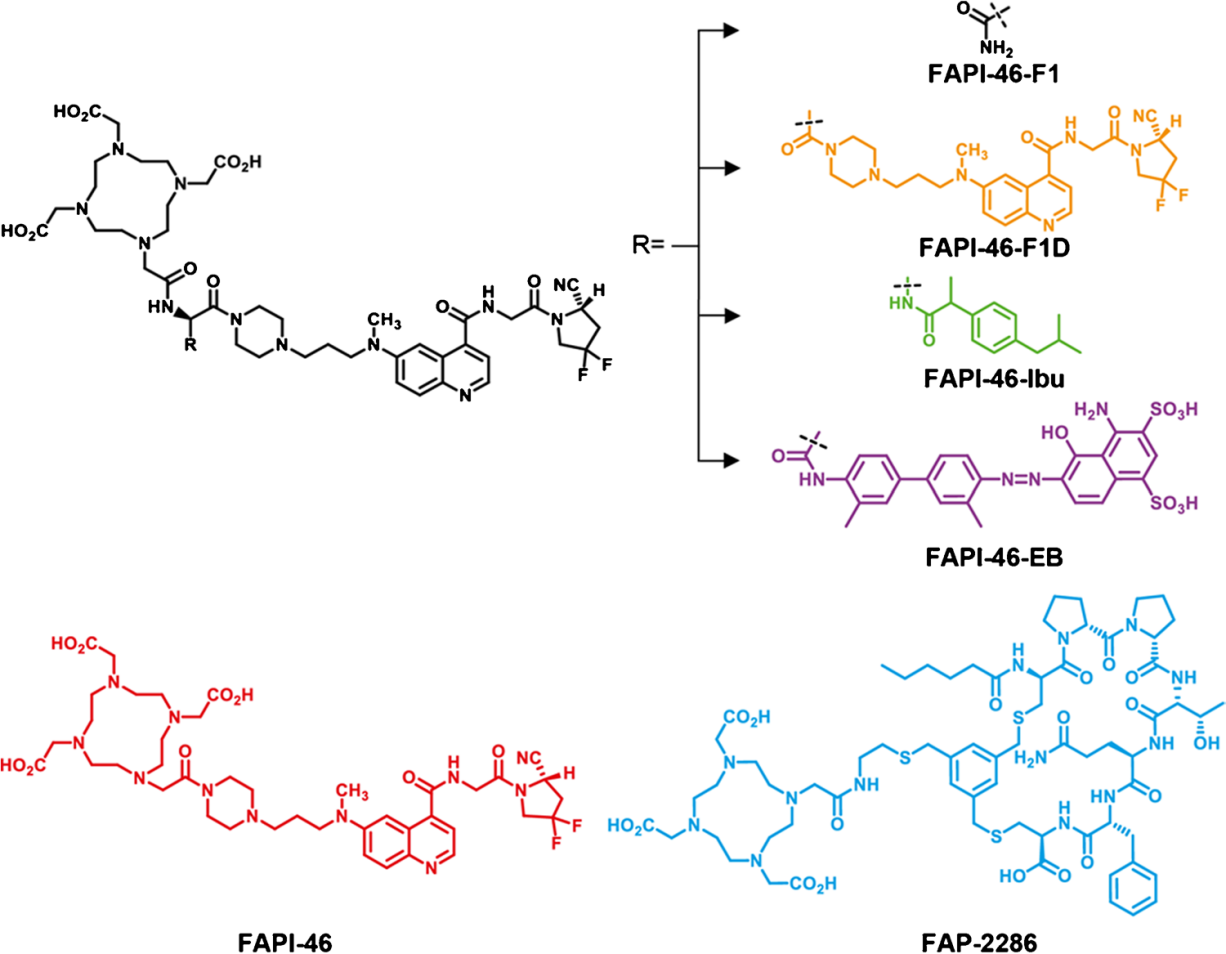
Chemical structures of the newly synthesized FAPI-46-based ligands, named FAPI-46-F1, FAPI-46-F1D, and the two albumin binder derivatives FAPI-46-Ibu and FAPI-46-EB, respectively. The structures of FAPI-46 and the peptide FAP-2286 are given for completion

FAPI-46, FAPI-46-F1 (a positive control), FAPI-46-F1D, FAPI-46-Ibu, FAPI-46-F1-EB, and FAP-2286, all conjugated to DOTA, were synthesized following well-established synthetic procedures [18, 19, 21]. The synthesis and the analytical data (HPLC and LC/MS) are provided in the “Supplementary information.” The structures are reported in Fig. 1.

^177^Lu-labeling was performed at different conditions, depending on the ligand (Supplementary Table 1). Lipophilicity was assessed by determining the distribution coefficient (log *D*_(pH 7.4)_) in 1-octanol/PBS (Supplementary Fig. 1**)**.

### Cell lines

HT-1080 and HEK-293 cells were transduced with the human FAP (hFAP). The production of lentiviral particles used for the transduction and the FACS gating strategy for the selection of monoclonal cell lines expressing FAP are provided in the “Supplementary information.” HT-1080.hFAP, a polyclonal cell line with heterogeneous and low FAP expression, and HEK-293.hFAP, a monoclonal cell line with high FAP expression (Supplementary Fig. 2), were used for the in vitro and in vivo evaluation. The two wild-type cell lines HT-1080.wt and HEK-293.wt were used to assess specificity.

Upon thawing, the cell lines were kept in culture in MEM supplemented with fetal bovine serum (10%) and penicillin-streptomycin (1%) at 37 °C and 5% CO_2_.

### Affinity determination and in vitro cellular uptake

The IC_50_ of all the ligands against isolated hFAP protein was assessed by an inhibition assay following published protocols [15, 22] (Supplementary Information and Supplementary Fig. 3).

Cellular uptake and distribution were assessed in FAP-positive cell lines at different times points (15 min, 1 h, and 4 h) after exposure to the radioligand at 37 °C. Wild-type cells were used to assess unspecific uptake. Details are provided in the “Supplementary information” (Supplementary Fig. 4 and Supplementary Table 2).

### Animal studies

All animal experiments were conducted in accordance with Swiss animal welfare laws and regulations under the license number 30515 granted by the Veterinary Office (Department of Health) of the Canton Basel-Stadt. Female athymic nude-Foxn1^nu^/Foxn1^+^ mice (Envigo, Netherlands), 4–6 weeks old, were used for generating FAP( +)/FAP( −) dual xenografts. Mice were implanted subcutaneously with 5–12 × 10^6^ FAP( +) and FAP( −) cells suspended in 100 μL PBS on the right and left shoulder (imaging studies) or right and left flank (biodistribution studies), respectively. The tumors were allowed to grow until reaching a volume of 100–200 mm^3^.

### SPECT/CT imaging studies

SPECT/CT images were acquired using a dedicated nanoSPECT/CT system (Bioscan, Mediso, Budapest, Hungary). Mice were injected intravenously via the tail vein with  ~ 9–15 MBq (500 pmol) of the radioligand and euthanized after 4 h. Details on image acquisition and reconstruction parameters are described in the “Supplementary information.”

### Biodistribution studies and AUC analysis

Mice were randomized (4–5/group), injected intravenously with the radioligand (100 µL/500 pmol/0.8–1 MBq), and euthanized at different time points (4 h, 24 h, 72 h, 120 h for HT-1080 and 4 h, 24 h, 72 h for HEK-293 xenografts, respectively) by CO_2_ asphyxiation. Organs of interest and blood were collected, rinsed of excess blood, blotted dry, weighed, and counted in a γ-counter. The samples were counted against a suitably diluted aliquot of the injected solution as the standard, and the results were expressed as the percentage of the injected activity per gram of tissue (%I.A./g) ± standard deviation (SD).

The area under the time-activity curves (AUC) in the tumors were generated from the biodistribution data and expressed as (%I.A./g)*h. AUC of tumor-to-critical-organs ratios were also generated. The calculations were performed using GraphPad Prism 9. Ninety-five percent confidence interval (95% CI) and statistical analysis (*p* values) of the AUC data are presented in the “Supplementary information.”

### Dosimetry

Non-decay corrected mice biodistribution data were used to generate time-activity curves for each radioligand. OLINDA/EXM 1.0 was used to integrate the fitted time-activity curves and to estimate the tumor doses and organ doses using the whole-body adult female model, as previously described [23]. For all calculations, the assumption was made that the mouse biodistribution, determined as the %I.A./organ, was the same as the human biodistribution.

## Results

### Synthesis, radiolabeling and lipophilicity

The FAP-binding moiety (*S*)-N-(2-(2-cyano-4,4-difluoropyrrolidin-1-yl)-2-oxoethyl)-6-(methyl(3-(piperazin-1-yl)propyl)amino)quinoline-4-carboxamide was coupled to aspartic acid in order to allow the modification of FAPI-46. The free carboxylic acid was used to add Evans Blue, or for dimerization of the binding moiety, to generate FAPI-46-F1-EB and FAPI-46-F1D, respectively. The positive control FAPI-46-F1 was synthesized by attaching asparagine to the FAP-binding moiety. DOTA was conjugated to the N-terminal. FAPI-46-Ibu was synthesized by solid-phase synthesis from beta-diamino propionic acid which was coupled to DOTA first and then to the FAP-binding moiety. The peptide FAP-2286 was synthesized by solid-phase peptide synthesis, following published procedures [19]. All the ligands were obtained in high purity. The structures are shown in Fig. 1.

All ^177^Lu-labeled ligands were prepared with apparent molar activities ranging from 8 up to 36 MBq/nmol, depending on the study, and radiochemical purity  ≥ 93%.

The new monomer [^177^Lu]Lu-FAPI-F1 was more hydrophilic than [^177^Lu]Lu-FAPI-46 (log *D* =  − 3.52 ± 0.04 vs  − 2.99 ± 0.04, respectively), and similar to [^177^Lu]Lu-FAP-2286 (− 3.43 ± 0.17). Dimerization introduced lipophilic characteristics similar to the conjugation of Evans Blue, while conjugation of ibuprofen led to the most lipophilic radioligand among all (log *D* =  − 2.28 ± 0.06,  − 2.65 ± 0.07, and  − 0.63 ± 0.13 for [^177^Lu]Lu-FAPI-F1D, [^177^Lu]Lu-FAPI-F1-EB, and [^177^Lu]Lu-FAPI-46-Ibu, respectively) (Supplementary Fig. 1).

### Affinity and cellular distribution

All the ligands showed excellent inhibition properties on hFAP (Supplementary Fig. 3). FAPI-46-F1D and FAPI-46-Ibu showed enhanced inhibitory activity (IC_50_ = 157.8 ± 14.5 and 39.4 ± 16.1 pM, respectively), while all the others showed reduced to very similar inhibitory activity (IC_50_ = 265.6 ± 35.9, 634.3 ± 102.3, and 247.6 ± 71.1 pM for FAPI-46-F1, FAPI-46-EB, and FAP-2286, respectively), compared to FAPI-46 (IC_50_ = 247.0 ± 17 pM).

All FAPI-46-based radioligands were internalized (35 up to 80% of the applied radioactivity at 4 h), with a minimum amount remaining on the cell surface (0.9 up to 4% at 4 h). [^177^Lu]Lu-FAP-2286 displayed different cellular distribution, with rather low internalization (20–30% of the applied radioactivity at 4 h), and high cell surface binding (50–60% at 4 h) (Supplementary Fig. 4A-B and Supplementary Table 2).

### SPECT/CT imaging

**Fig. 2:**
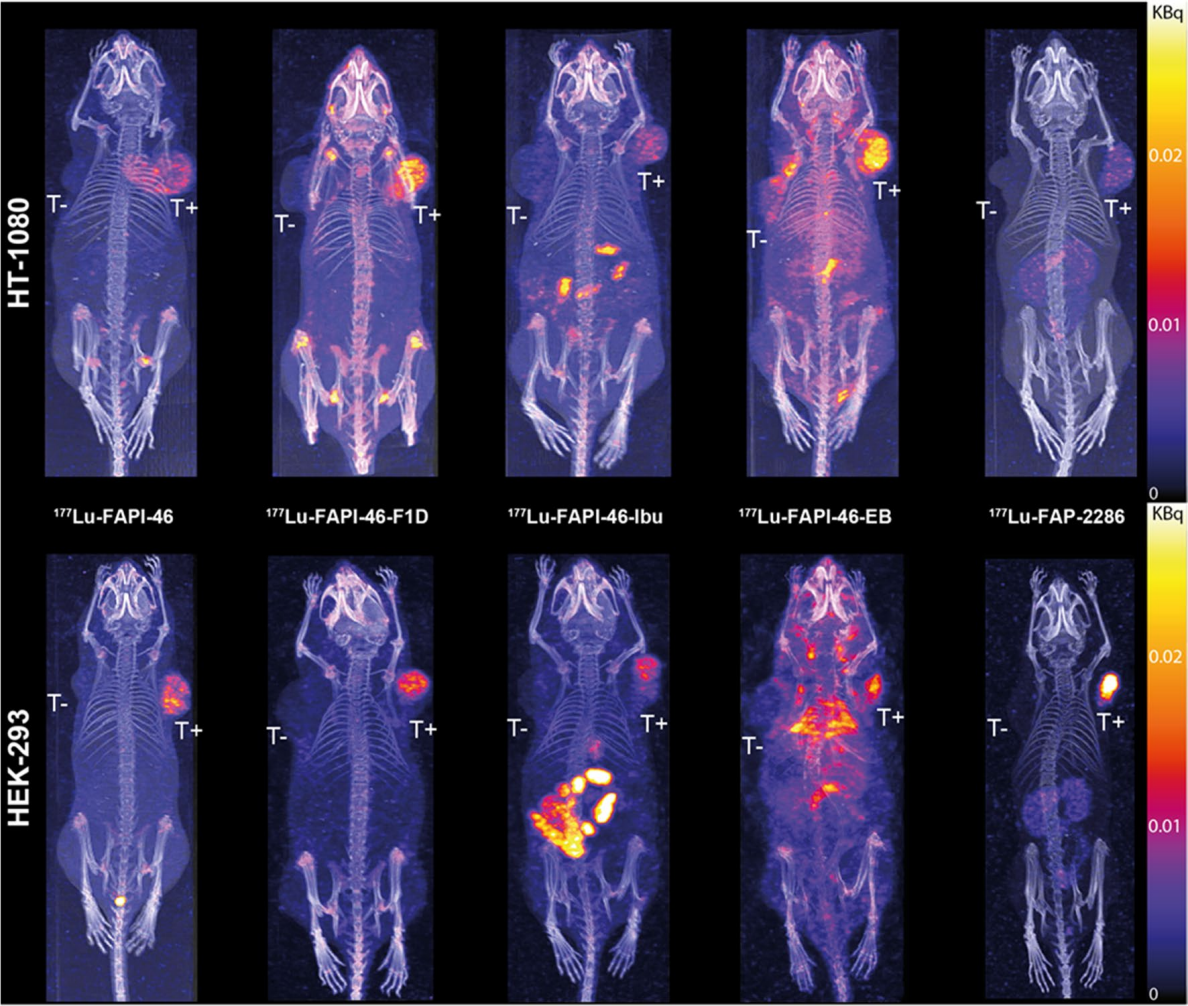
SPECT/CT images of [^177^Lu]Lu-FAPI-46, [^177^Lu]Lu-FAPI-46-F1D, [^177^Lu]Lu-FAPI-46-Ibu, [^177^Lu]Lu-FAPI-46-EB, and [^177^Lu]Lu-FAP-2286 (500 pmol/9–15 MBq) at 4 h p.i. in mice bearing low FAP-expressing model HT-1080.hFAP and the wild type HT-1080.wt (top) or high FAP-expressing model HEK-293.hFAP and the wild type HEK-293.wt (bottom) dual tumors. All radioligands accumulated in the FAP-expressing tumors to different extend and exhibited distinct differences in their total body distribution. T +  = FAP-positive tumor, T −  = FAP-negative tumor

The visual assessment of SPECT/CT images at 4 h p.i. (Fig. 2) showed accumulation of all radioligands in the FAP-expressing tumors, with no accumulation in the wild-type tumors, with the exception of the [^177^Lu]Lu-FAPI-46-EB in the HT1080.wt tumors. In the HT-1080-xenografts, [^177^Lu]Lu-FAPI-46-F1D showed the highest FAP-tumor uptake, while [^177^Lu]Lu-FAP-2286 presented the lowest, thought the best tumor-to-background contrast. In the HEK-293-xenografts, [^177^Lu]Lu-FAP-2286 presented the highest FAP-tumor uptake, together with the best tumor-to-background contrast. In both tumor models, [^177^Lu]Lu-FAPI-46-EB had the highest background. Quantification of the remaining activity in the body at 4 h p.i. (Supplementary Table 3) verified the fastest washout of the [^177^Lu]Lu-FAP-2286 and the highest body retention of [^177^Lu]Lu-FAPI-46-EB (6–8% vs 80–90% of the injected activity remained in the body at 4 h p.i., respectively), among all radioligands.

### Biodistribution and AUC

Biodistribution results generated by gamma-counting harvested organs are provided in Tables 1, 2, 3, 4, and 5. Extended biodistribution data are provided in Supplementary Tables 4–8. Because [^177^Lu]Lu-FAPI-46-F1 did not present any advantage compared to [^177^Lu]Lu-FAPI-46 at 4 h and 24 h p.i. (Supplementary Table 9) the biodistribution was not further investigated.

**Table 1:** [^177^Lu]Lu-FAPI-46 biodistribution results in HT-1080 and HEK-293 xenografts expressed as mean of the % injected activity per gram of tissue (%I.A./g) ± standard deviation (SD) (*n* = 4–5/group)

Organ	HT-1080 xenografts			HEK-293 xenografts		
	4 h	24 h	72 h	4 h	24 h	72 h
Blood	0.43 ± 0.04	0.03 ± 0.01	0.00 ± 0.00	0.65 ± 0.08	0.03 ± 0.01	0.00 ± 0.00
Lung	0.29 ± 0.03	0.05 ± 0.02	0.03 ± 0.00	0.41 ± 0.08	0.04 ± 0.01	0.01 ± 0.00
Liver	0.29 ± 0.02	0.21 ± 0.06	0.24 ± 0.05	0.35 ± 0.05	0.19 ± 0.07	0.10 ± 0.04
Kidney	1.19 ± 0.34	0.59 ± 0.05	0.39 ± 0.12	1.33 ± 0.19	0.38 ± 0.06	0.15 ± 0.10
Muscle	0.58 ± 0.38	0.09 ± 0.05	0.04 ± 0.03	0.49 ± 0.15	0.08 ± 0.03	0.01 ± 0.00
Femur	2.52 ± 0.38	0.61 ± 0.14	0.73 ± 0.08	2.22 ± 0.58	0.55 ± 0.22	0.19 ± 0.06
hFAP-tumor	9.63 ± 1.79	2.68 ± 0.74	1.58 ± 0.37	10.34 ± 4.55	3.39 ± 1.65	0.44 ± 0.11
wt-tumor	1.02 ± 0.18	0.35 ± 0.13	0.17 ± 0.04	1.40 ± 0.39	0.28 ± 0.07	0.05 ± 0.01

**Table 2:** [^177^Lu]Lu-FAPI-46-F1D biodistribution results HT-1080 and HEK-293 xenografts expressed as mean of the % injected activity per gram of tissue (%I.A./g) ± standard deviation (SD) (*n* = 4–6/group)

Organ	HT-1080			HEK-293		
	4 h	24 h	72 h	4 h	24 h	72 h
Blood	1.21 ± 0.22	0.60 ± 0.05	0.04 ± 0.01	1.51 ± 0.28	0.59 ± 0.08	0.05 ± 0.01
Lung	0.87 ± 0.21	0.53 ± 0.01	0.18 ± 0.03	0.93 ± 0.13	0.55 ± 0.07	0.25 ± 0.04
Liver	0.67 ± 0.16	1.37 ± 0.04	1.23 ± 0.27	0.83 ± 0.25	1.35 ± 0.15	1.66 ± 0.31
Kidney	1.25 ± 0.08	1.02 ± 0.16	0.87 ± 0.20	1.28 ± 0.19	0.94 ± 0.06	0.73 ± 0.06
Muscle	1.35 ± 0.43	0.88 ± 0.10	0.41 ± 0.21	1.05 ± 0.27	0.80 ± 0.11	0.41 ± 0.06
Femur	4.91 ± 0.75	2.89 ± 0.08	2.02 ± 0.32	5.19 ± 0.86	3.04 ± 0.27	2.09 ± 0.23
hFAP-tumor	10.47 ± 2.47	6.42 ± 0.89	3.57 ± 0.73	17.16 ± 4.63	5.89 ± 1.29	1.43 ± 0.20
wt-tumor	2.26 ± 0.32	2.57 ± 0.42	1.14 ± 0.38	3.27 ± 1.89	1.99 ± 0.26	1.41 ± 0.38

**Table 3:** [^177^Lu]Lu-FAPI-46-Ibu biodistribution results HT-1080 and HEK-293 xenografts expressed as mean of the % injected activity per gram of tissue (%I.A./g) ± standard deviation (SD) (*n* = 4/group)

Organ	HT-1080			HEK-293		
	4 h	24 h	72 h	4 h	24 h	72 h
Blood	1.07 ± 0.10	0.14 ± 0.03	0.01 ± 0.00	1.00 ± 0.18	0.10 ± 0.01	0.00 ± 0.00
Lung	0.83 ± 0.07	0.21 ± 0.03	0.07 ± 0.02	1.88 ± 2.29	0.15 ± 0.01	0.04 ± 0.01
Liver	0.96 ± 0.08	1.02 ± 0.70	0.50 ± 0.02	0.75 ± 0.11	0.58 ± 0.35	0.35 ± 0.04
Kidney	1.80 ± 0.15	1.15 ± 0.19	0.54 ± 0.11	1.33 ± 0.17	0.81 ± 0.11	0.25 ± 0.03
Muscle	0.97 ± 0.25	0.43 ± 0.28	0.12 ± 0.07	0.87 ± 0.36	0.28 ± 0.08	0.07 ± 0.03
Femur	3.62 ± 0.04	1.12 ± 0.12	0.51 ± 0.09	2.46 ± 0.88	0.81 ± 0.11	0.33 ± 0.08
hFAP-tumor	8.40 ± 1.97	5.09 ± 0.64	1.55 ± 0.58	5.03 ± 2.84	3.14 ± 0.49	0.88 ± 0.40
wt-tumor	1.96 ± 0.36	1.00 ± 0.25	0.28 ± 0.05	1.86 ± 1.25	0.52 ± 0.11	0.18 ± 0.05

**Table 4:** [^177^Lu]Lu-FAPI-46-EB biodistribution results HT-1080 and HEK-293 xenografts expressed as mean of the % injected activity per gram of tissue (%I.A./g) ± standard deviation (SD) (*n* = 4/group)

Organ	HT-1080			HEK-293		
	4 h	24 h	72 h	4 h	24 h	72 h
Blood	11.59 ± 0.46	5.47 ± 0.85	1.63 ± 0.19	13.16 ± 2.19	6.85 ± 0.61	1.18 ± 0.05
Lung	5.45 ± 0.23	3.27 ± 0.60	1.64 ± 0.08	6.45 ± 1.19	3.98 ± 0.44	1.45 ± 0.06
Liver	2.80 ± 0.26	2.66 ± 0.31	2.78 ± 0.48	3.06 ± 0.36	3.05 ± 0.12	2.29 ± 0.23
Kidney	4.33 ± 0.12	5.02 ± 0.59	5.97 ± 0.49	4.70 ± 1.03	6.67 ± 0.67	4.86 ± 0.72
Muscle	1.31 ± 0.07	1.17 ± 0.13	0.85 ± 0.19	1.61 ± 0.17	1.39 ± 0.27	0.59 ± 0.25
Femur	2.35 ± 0.24	2.09 ± 0.21	1.34 ± 0.13	2.43 ± 0.26	2.51 ± 0.30	1.08 ± 0.24
hFAP-tumor	5.02 ± 0.29	5.44 ± 1.03	4.16 ± 0.19	12.58 ± 2.83	16.69 ± 1.01	6.75 ± 1.06
wt-tumor	3.70 ± 0.72	3.56 ± 0.76	3.75 ± 1.02	3.75 ± 0.40	4.25 ± 0.47	2.10 ± 0.07

**Table 5:** [^177^Lu]Lu-FAP-2286 biodistribution results HT-1080 and HEK-293 xenografts expressed as mean of the % injected activity per gram of tissue (%I.A./g) ± standard deviation (SD) (*n* = 4/group)

Organ	HT-1080			HEK-293		
	4 h	24 h	72 h	4 h	24 h	72 h
Blood	0.03 ± 0.01	0.00 ± 0.00	0.00 ± 0.00	0.04 ± 0.01	0.00 ± 0.00	0.00 ± 0.00
Lung	0.08 ± 0.01	0.03 ± 0.01	0.01 ± 0.00	0.07 ± 0.00	0.02 ± 0.00	0.01 ± 0.00
Liver	0.11 ± 0.02	0.08 ± 0.01	0.05 ± 0.01	0.11 ± 0.01	0.09 ± 0.00	0.03 ± 0.01
Kidney	5.15 ± 0.65	3.62 ± 0.51	1.58 ± 0.51	3.98 ± 0.42	2.33 ± 0.48	0.72 ± 0.22
Muscle	0.04 ± 0.01	0.02 ± 0.01	0.01 ± 0.00	0.06 ± 0.03	0.02 ± 0.01	0.01 ± 0.01
Femur	0.15 ± 0.05	0.10 ± 0.01	0.16 ± 0.06	0.27 ± 0.04	0.20 ± 0.01	0.04 ± 0.02
hFAP-tumor	3.42 ± 1.06	1.67 ± 0.50	0.64 ± 0.22	22.99 ± 3.13	13.49 ± 1.95	4.05 ± 0.99
wt-tumor	0.10 ± 0.01	0.08 ± 0.01	0.04 ± 0.01	0.28 ± 0.13	0.13 ± 0.05	0.02 ± 0.02

In the low FAP-expressing model HT-1080.hFAP, the tumor uptake at 4 h p.i. (peak uptake) followed the order [^177^Lu]Lu-FAPI-46-F1D ≥ [^177^Lu]Lu-FAPI-46 ≥ [^177^Lu]Lu-FAPI-46-Ibu > [^177^Lu]Lu-FAPI-46-EB > [^177^Lu]Lu-FAP-2286 (10.47 vs 9.63 vs 8.40 vs 5.02 vs 3.42%I.A./g). However, after 72 h, only the [^177^Lu]Lu-FAPI-46-EB retained highly in the tumor (83%), followed by [^177^Lu]Lu-FAPI-46-F1D (34%). Less than 20% retained in the tumor for the other three radioligands. Interestingly, [^177^Lu]Lu-FAPI-46-EB showed high and persistent accumulation in HT1080.wt tumors; e.g., at 4 h p.i. 74% of the uptake found in HT1080.hFAP was determined in the HT1080.wt. This was  ~ 20% for [^177^Lu]Lu-FAPI-46-F1D and [^177^Lu]Lu-FAPI-46-Ibu, significantly lower for [^177^Lu]Lu-FAPI-46 (~ 10%), and almost negligible for [^177^Lu]Lu-FAP-2286 (~ 3%).

In the high FAP-expressing model HEK-293.hFAP, the tumor uptake at 4 h p.i. (peak uptake) followed another order: [^177^Lu]Lu-FAP-2286 > [^177^Lu]Lu-FAPI-46-F1D > [^177^Lu]Lu-FAPI-46-EB ≥ [^177^Lu]Lu-FAPI-46 > [^177^Lu]Lu-FAPI-46-Ibu (22.99 vs 17.16 vs 12.58 vs 10.34 vs 5.03%I.A./g). After 72 h, [^177^Lu]Lu-FAPI-46-EB showed the highest tumor retention (54%), followed by [^177^Lu]Lu-FAP-2286 and [^177^Lu]Lu-FAPI-46-Ibu (18%). [^177^Lu]Lu-FAPI-46-F1D and [^177^Lu]Lu-FAPI-46 retained in the tumor only by  ~ 8% and  ~ 4%, respectively. Regarding specificity, [^177^Lu]Lu-FAPI-46-EB and [^177^Lu]Lu-FAPI-46-Ibu showed the highest nonspecific binding; e.g. at 4 h p.i. 30–40% of the uptake found in HEK293.hFAP was determined in HEK293.wt. This was 19% for [^177^Lu]Lu-FAPI-46-F1D,  ~ 14% for [^177^Lu]Lu-FAPI-46, and only 1% for [^177^Lu]Lu-FAP-2286.

Concerning total body distribution and pharmacokinetics, the two monomers [^177^Lu]Lu-FAPI-46 and [^177^Lu]Lu-FAP-2286 showed significantly lower background activity. [^177^Lu]Lu-FAP-2286 presented the lowest background, with the exception the kidney uptake. Among the other three radioligands, [^177^Lu]Lu-FAPI-46-Ibu showed lower background (still higher than the monomers), followed by [^177^Lu]Lu-FAPI-46-F1D. [^177^Lu]Lu-FAPI-46-EB showed the highest background activity at all investigated time points.

**Fig. 3:**
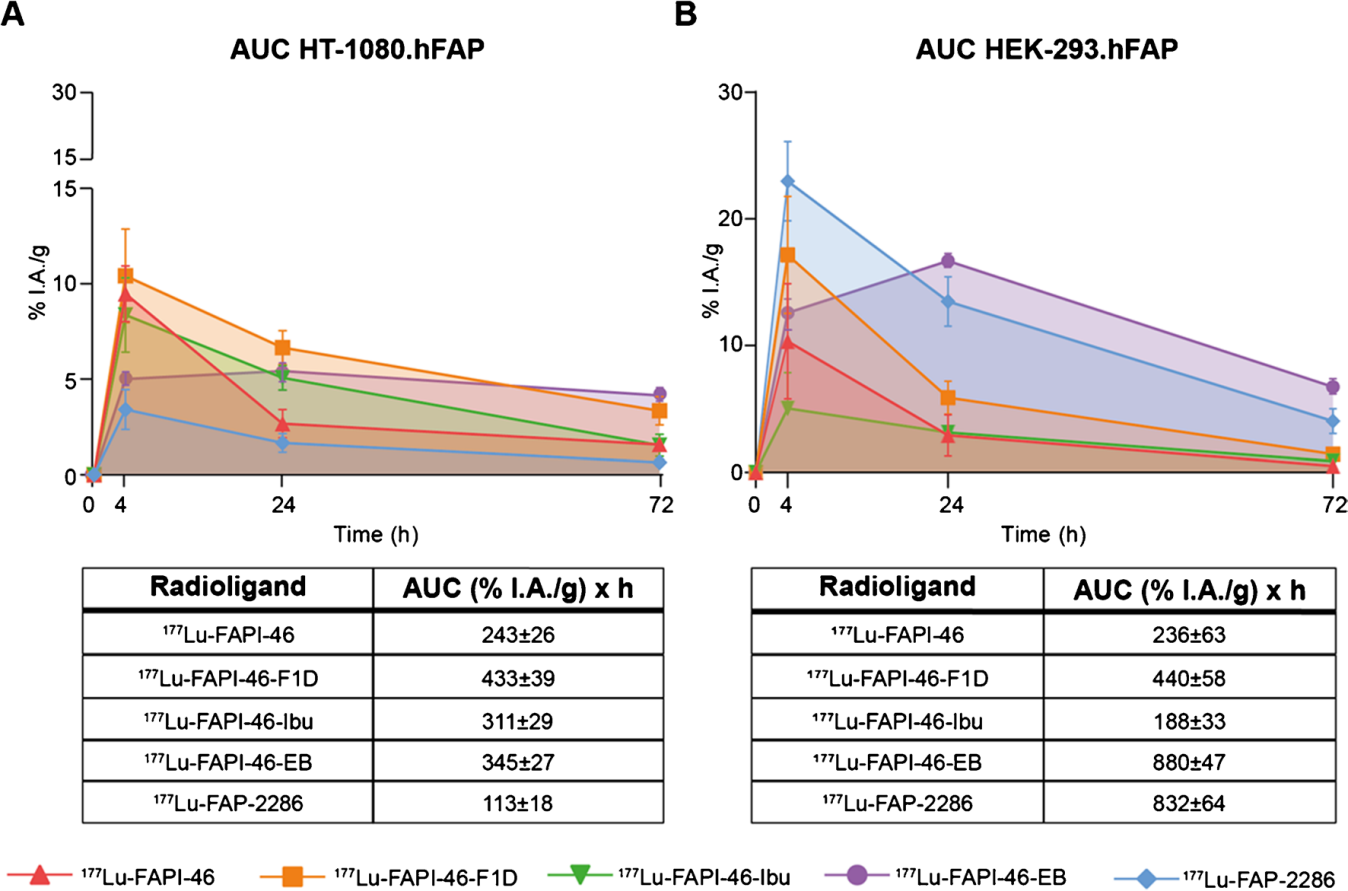
The area under the time activity curve (AUC) in HT-1080.hFAP **A** and HEK-293.hFAP **B** expressed as mean ± standard deviation. These pharmacokinetic data were generated from serial independent biodistribution experiments performed 4, 24, and 72 h post injection. While [^177^Lu]Lu-FAPI-46, [^177^Lu]Lu-FAPI-46-F1D, and [^177^Lu]Lu-FAPI-46-Ibu showed similar AUC values in the two tumor models, [^177^Lu]Lu-FAPI-46-EB and [^177^Lu]Lu-FAP-2286 exhibited significantly higher AUC values in the HEK-293.hFAP tumor (high FAP expression), compared with the HT-1080.hFAP (low FAP expression)

The AUC of the tumor uptake over time was assessed from the biodistribution data as a surrogate of the radiation dose delivered to the tumors (Fig. 3A and B and Supplementary Table 10). In the low FAP-expressing tumors, [^177^Lu]Lu-FAPI-46-F1D and the two albumin binder conjugates, [^177^Lu]Lu-FAPI-46-Ibu and [^177^Lu]Lu-FAPI-46-EB, presented the highest AUCs. In the high FAP-expressing tumors, [^177^Lu]Lu-FAPI-46-EB and [^177^Lu]Lu-FAP-2286 had the highest tumor AUC. Interestingly, [^177^Lu]Lu-FAPI-46-EB showed 2.6-fold increase in the high vs low FAP-expressing tumors (880 vs 345%I.A./g*h, respectively) and [^177^Lu]Lu-FAP-2286 an increase of 7.4-fold (832 *vs* 113%I.A./g*h, respectively). No difference was observed in the AUC of [^177^Lu]Lu-FAPI-46 and [^177^Lu]Lu-FAPI-46-F1D between the two tumor models.

**Fig. 4:**
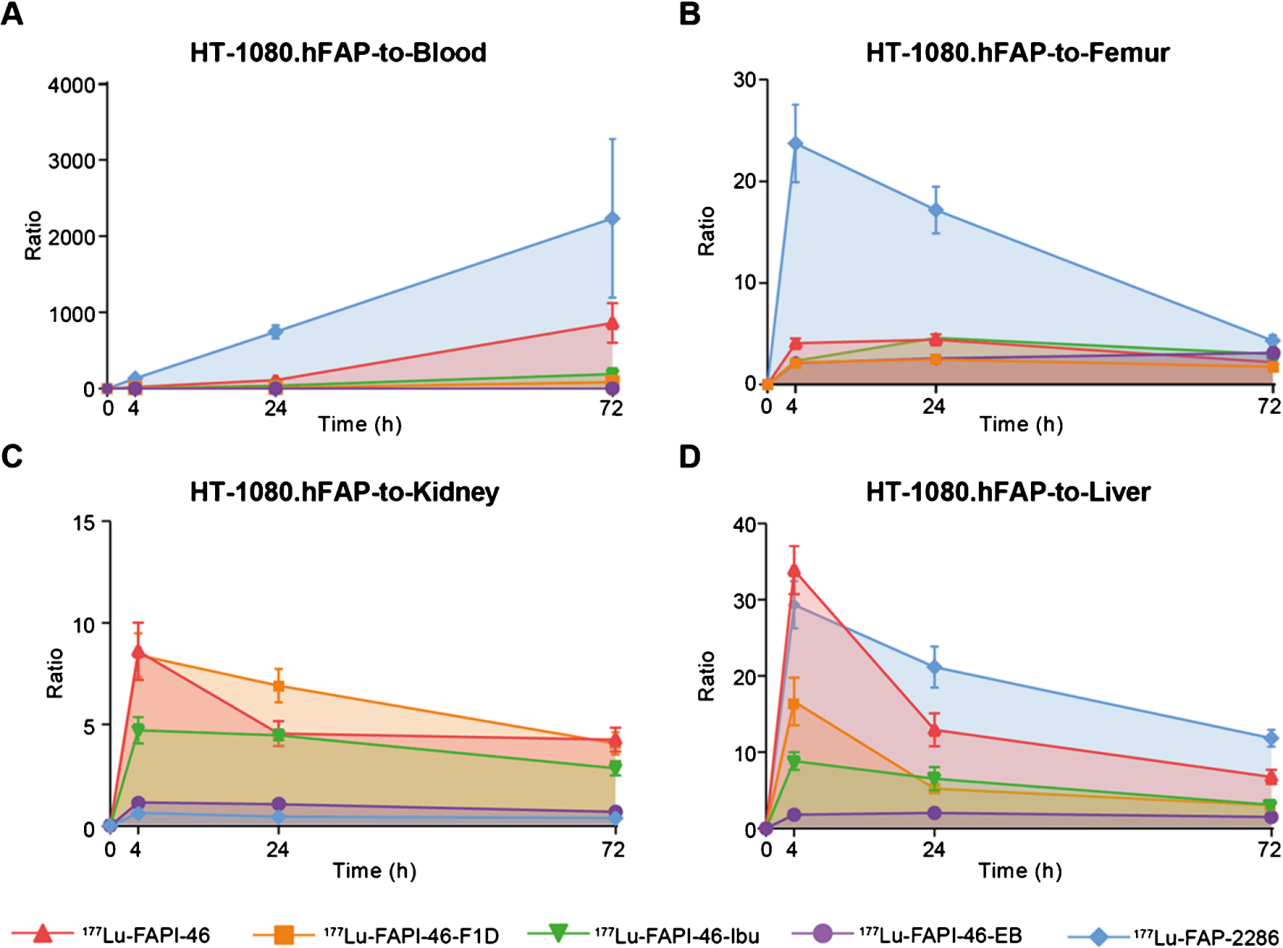
The area under the curve (AUC) of **A** tumor-to-blood, **B** tumor-to-femur, **C** tumor-to-kidney, and **D** tumor-to-liver ratio of the radioligands in the low FAP-expressing HT-1080.hFAP xenografts, expressed as mean ± standard deviation. With the exception of the tumor-to-kidney ratio over time, [^177^Lu]Lu-FAP-2286 presented the most favorable AUC of the tumor-to-blood, tumor-to-femur, and tumor-to-liver. Tumor-to-kidney AUCs were favorable for [^177^Lu]Lu-FAPI-46- F1D, [^177^Lu]Lu-FAPI-46-Ibu, and [^177^Lu]Lu-FAPI-46, compared to the other two radioligands

**Fig. 5:**
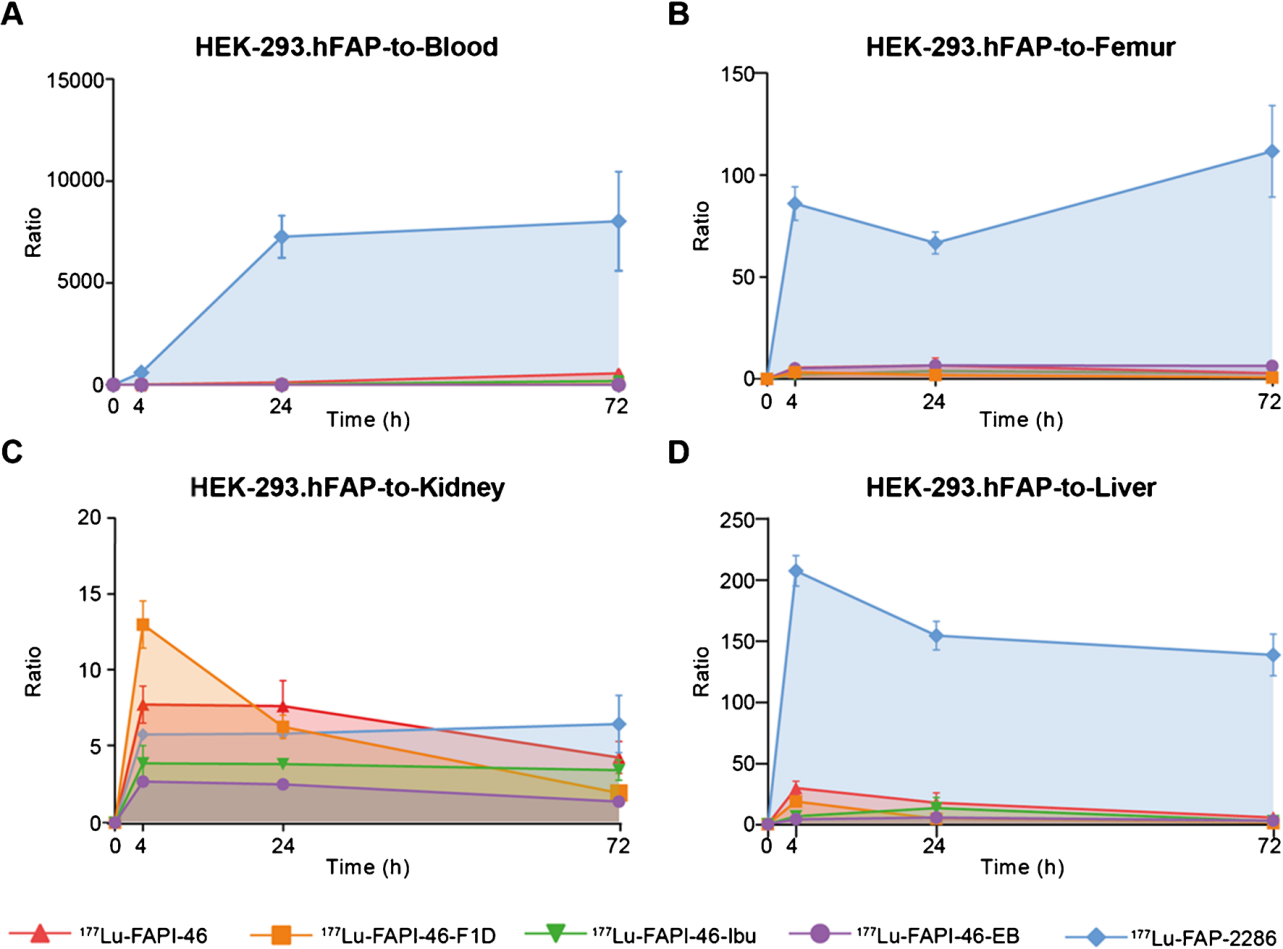
Area under the curve (AUC) of **A** tumor-to-blood, **B** tumor-to-femur, **C** tumor-to-kidney, and **D** tumor-to-liver ratio of the radioligands in the high FAP-expressing HEK-293.hFAP xenografts, expressed as mean ± standard deviation. [^177^Lu]Lu-FAP-2286 presented the most favorable tumor-to-blood, tumor-to-femur, and tumor-to-liver ratios over time. Tumor-to-kidney AUC values were in a comparable range for all radioligands, with the exception of the [^177^Lu]Lu-FAPI-46-EB that showed a significantly lower AUC

The AUCs of the tumor-to-critical-organs ratios were assessed as indicators of the therapeutic index (Figs. 4 and 5). With the exception of the tumor-to-kidneys ratio, [^177^Lu]Lu-FAP-2286 presented the most favorable tumor-to-critical-organs ratios over time in both tumor models, as indicated by the AUC of tumor-to-blood, tumor-to-liver, and tumor-to-femur ratios.

### Dosimetry

The dosimetry estimates for the critical organs and the FAP-expressing tumors are reported in Table 6. The low FAP-expressing tumors received the highest radiation dose from [^177^Lu]Lu-FAPI-46-F1D, [^177^Lu]Lu-FAPI-46-Ibu, and [^177^Lu]Lu-FAPI-46-EB (3.67, 3.64, and 7.52 mGy/MBq, respectively), while the high FAP-expressing tumors received the highest dose from [^177^Lu]Lu-FAP-2286 and [^177^Lu]Lu-FAPI-46-EB (6.76 and 11.3 mGy/MBq, respectively). Regarding the critical organs, the absorbed dose in the red marrow was highest for [^177^Lu]Lu-FAPI-46-EB, followed by [^177^Lu]Lu-FAPI-46-Ibu (2.79E-02 and 6.50E-03 mGy/MBq, respectively), while [^177^Lu]Lu-FAPI-46-F1D delivered 11 and 2.6 times lower dose, respectively, though higher than [^177^Lu]Lu-FAPI-46 and [^177^Lu]Lu-FAP-2286. The absorbed dose to the kidneys was highest for [^177^Lu]Lu-FAPI-46-EB (3.71E + 00 mGy/MBq), followed by [^177^Lu]Lu-FAP-2286 and [^177^Lu]Lu-FAPI-46-F1D that delivered doses of one order of magnitude lower (2.04E-01 and 1.11E-01 mGy/MBq, respectively). Among all radioligands, the lowest dose to the kidneys was delivered by [^177^Lu]Lu-FAPI-46 (4.69E-02 mGy/MBq) and the lowest dose to the red marrow and the liver was delivered by [^177^Lu]Lu-FAP-2286 (1.78E-04 and 4.21E-03 mGy/MBq, respectively).

**Table 6:** Radiation dosimetry estimation in tumors and critical organs expressed as mean absorbed dose (mGy/MBq). Results were obtained with OLINDA/EXM 1.0 by integrating the fitted time-activity curves

Organ	[^177^Lu]Lu-FAPI-46	[^177^Lu]Lu-FAPI-46-F1D	[^177^Lu]Lu-FAPI-46-Ibu	[^177^Lu]Lu-FAPI-46-EB	[^177^Lu]Lu-FAP-2286
Kidneys	4.69E-02	1.11E-01	6.75E-02	3.71E+00	2.04E-01
Liver	3.58E-02	1.18E-01	4.25E-02	3.23E-01	4.21E-03
Red marrow	4.05E-04	2.53E-03	6.50E-03	2.79E-02	1.78E-04
HT-1080.hFAP tumor	1.73E+00	3.67E+00	3.64E+00	7.25E+00	8.62E-01
HEK-293.hFAP tumor	1.72E+00	2.31E+00	1.75E+00	1.13E+01	6.76E+00

## Discussion

The therapeutic value of FAP-targeting radioligands is harmed mainly due to their short tumor residence time [7, 11, 24]. Three different strategies have been proposed for prolonging tumor residence time: (a) multimerization of the FAP-binding moiety [15, 16, 25], (b) conjugation of an albumin binder [17, 18, 28,26-], and (c) peptide-based structures as an alternative to small molecules [19, 20]. Nevertheless, a direct comparison among them is still missing. We, therefore, synthesized and tested head-to-head a panel of FAP radioligands representing all the above-mentioned strategies, including the new albumin-binder conjugate [^177^Lu]Lu-FAPI-46-Ibu. Our aim was to identify the strengths and limitations of the different strategies that need to be considered in the development of FAP-targeting radiotherapeutics.

In vitro, all radioligands showed very high affinity to hFAP, with a certain variation among them, and IC_50_ values in the picomolar range. This allowed a fair comparison in vivo regarding FAP-targeting. Differences were observed in their cellular distribution. All [^177^Lu]Lu-FAPI-46-based radioligands were almost entirely internalized, while [^177^Lu]Lu-FAP-2286 remained mainly on the cell surface.

Focusing on the first strategy of dimerization, [^177^Lu]Lu-FAPI-46-F1D presented a higher and more persistent uptake in the tumor, compared to the monomer [^177^Lu]Lu-FAPI-46, independent of the tumor model. This is in line with the recently published studies on FAP-targeting dimers, such as BiOncoFAP [15], DOTAGA.(SA.FAPi)_2_ [16, 29, 30], DOTA-2P(FAPi)_2_ [25, 31], and ND-bisFAPI [14]. Undoubtfully, this observation supports the use of multimers for FAP-targeting radiotherapeutics per se. Evidently, total body distribution and pharmacokinetics are just as important as tumor uptake. In our study, it was shown that dimerization doubled the radiation dose delivered to the tumor, but also increased the dose to non-targeted organs, especially in blood, femur, liver, and kidneys, and the overall background activity, suggesting higher toxicity. The first-in-human dosimetry study of [^177^Lu]Lu-DOTAGA.(SA.FAPi)_2_ vs the monomer [^177^Lu]Lu-DOTAGA.SA.FAPi demonstrated a significantly longer tumor retention, accompanied by a significantly higher whole-body effective half-life and uptake in healthy organs (e.g., colon and kidneys) [16]. No data about the therapeutic efficacy are available so far [16].

Focusing on the second strategy of the albumin binder conjugation, our study indicated that the outcome heavily depends on the albumin binder moiety of choice. [^177^Lu]Lu-FAPI-46-EB was found to be highly concentrated in the blood, while [^177^Lu]Lu-FAPI-46-Ibu presented a much faster clearance (1/10th compared with [^177^Lu]Lu-FAPI-46-EB at 4 h p.i. and even less at later time points). A recent study on FAPI-04 conjugates with the albumin binders 4-(*p*-iodophenyl)butyric acid ([^177^Lu]Lu-TEFAPI-06) and Evans Blue ([^177^Lu]Lu-TEFAPI-07) also indicated significant differences between the two moieties [17]. The blood concentration of the [^177^Lu]Lu-TEFAPI-06 was higher compared with [^177^Lu]Lu-TEFAPI-07 (e.g., 12.3 vs 5.64%IA/g at 24 h p.i.), but the tumor uptake and retention was the same for both. Our biodistribution data with [^177^Lu]Lu-FAPI-46-EB are in line with the EB conjugate [^177^Lu]Lu-TEFAPI-07 [17]. In our study, between the two albumin binders, the new [^177^Lu]Lu-FAPI-46-Ibu did not provide any advantage over [^177^Lu]Lu-FAPI-46, while [^177^Lu]Lu-FAPI-46-EB had serious limitations regarding specificity and total body radiation exposure. Overall, none of the two conjugates showed clear advantages over [^177^Lu]Lu-FAPI-46. Similar results were found with FAPI-02 EB conjugates via PEGylation vs unmodified FAPI-02 [18].

The third strategy of using peptides as an alternative to small molecules showed to be the best choice in tumors highly expressing FAP. [^177^Lu]Lu-FAP-2286 had the highest and also durable uptake in HEK293.hFAP tumors, and the lowest uptake in healthy organs, with the exception of the kidneys. Our results confirmed previous findings with [^177^Lu]Lu-FAP-2286 regarding biodistribution and tumor retention in HEK-293.hFAP xenografts [19]. Surprisingly, in the HT-1080.hFAP xenografts the tumor uptake of [^177^Lu]Lu-FAP-2286 was significantly lower, being the lowest among all studied radioligands. Nevertheless, the AUC of the tumor-to-critical-organs ratio was in favor of [^177^Lu]Lu-FAP-2286, despite the lowest tumor uptake. The exception remained the tumor-to-kidneys ratio, rendering kidneys the critical organ. However, the estimated absorbed dose of [^177^Lu]Lu-FAP-2286 to the kidneys was in the same level as [^177^Lu]Lu-DOTA-TATE (2.04E-01 vs 2.13E-01 mGy/MBq), assessed by the same methodology [23], while its red marrow dose was lower (1.78E-04 vs 1.25E-03 mGy/MBq, respectively). Preliminary human data with [^177^Lu]Lu-FAP-2286 indicated that the delivered dose to the whole body, bone marrow and kidneys were comparable to that of Pluvicto and Lutathera ([^177^Lu]Lu-DOTA-TATE) [20]. The same study showed that the tumor half-life of [^177^Lu]Lu-FAP-2286 is shorter than the above-mentioned approved radiotherapeutics, even though longer compared to the FAPI-based small molecules [20].

Last, but not least, we tried to understand the discrepancy between HT-1080.hFAP and HEK-293.hFAP on the in vivo uptake of [^177^Lu]Lu-FAP-2286. Our initial hypothesis was that a saturation level was reached in HT-1080.hFAP tumors with the injected mass of 500 pmol used in the study, given the low expression level of FAP. We, therefore, evaluated the biodistribution of [^177^Lu]Lu-FAP-2286 and of [^177^Lu]Lu-FAPI-46 in HT-1080.hFAP xenografts using tenfold less amount (Supplementary Table 11). The results using 50 pmol instead of 500 pmol indicated that no saturation was reached. To determine the saturation effect on the two tumors, an ex vivo blocking study was performed for [^177^Lu]Lu-FAP-2286 and [^177^Lu]Lu-FAPI-46 in both tumor models. In each case, 60-fold excess of the non-labeled ligand was administered 5 min before the injection of the corresponding radioligand. While in the HT-1080.hFAP tumors a complete inhibition of the radioligand uptake was observed, in the high-expressing FAP cell line HEK-293.hFAP tumors the inhibition was lower, still significantly lower when compared to the radioligand uptake without the blocking (Supplemental Fig. 5 and 6). These results indicate that the HEK-293.hFAP model has available amounts of hFAP that require more than 30.5 nmol of FAP ligands to be completely occupied. The in vitro autoradiography performed on HT-1080.hFAP and HEK-293.hFAP tumor slides after incubation with [^177^Lu]Lu-FAPI-46 and [^177^Lu]Lu-FAP-2286 with and without the presence of 10,000-fold excess of the non-labeled ligand (Supplemental Fig. 7) corroborated these results. In addition, the autoradiography confirmed the difference observed in vivo between the [^177^Lu]Lu-FAP-2286 and [^177^Lu]Lu-FAPI-46 in the two tumor models. Our second hypothesis was that the two classes of the studied ligands, FAPI small molecules and a cyclic peptide, may present different binding sites as they are structurally very different. To test this hypothesis, we performed some preliminary in vitro experiments on cell membranes. We observed lower blocking efficiency when FAPI-46 was used to block the binding of [^177^Lu]Lu-FAP-2286, compared to its efficiency to block [^177^Lu]Lu-FAPI-46 (data not shown). This is an indication that the two ligands might present different binding sites. However, further and more sophisticated experiments have to be designed for testing this hypothesis.

The presented results on two cell lines with distinct expression and homogeneity levels of FAP (polyclonal *vs* high-expressing monoclonal, Supplementary Fig. 3) underlined the importance of the tumor model in assessing FAP-targeting ligands. Different target density on the cell surface may have a profound impact on the receptor occupancy, affecting the total uptake of the radioligand [32]. Moreover, since FAP is known to be active upon homodimerization, a higher receptor density may promote oligomerization, affecting the radioligand binding [33]. Furthermore, it is known that the glycosylation pattern can vary among different cell lines expressing the same protein, rendering the binding site of radioligands less accessible [34]. Complementary to our second hypothesis, we may speculate that homo/oligo-merization and/or glycosylation pattern is more relevant for the binding of the FAP-targeting peptide-based structures than the quinoline-based small-molecule inhibitors. This might explain why the uptake of the [^177^Lu]Lu-FAP-2286 was significantly impacted by the FAP-expression level and density, which was not the case for the FAPI-46-based radioligands. Nevertheless, as far as we know, no data are available in the literature to support this hypothesis. Finally, using cell lines with distinct characteristics and FAP expression levels may elucidate the interactions of structurally different radioligands with FAP.

To the best of our knowledge, this is the only study so far providing a fair comparison among the different structural designs. We choose representative radioligands from each strategy with very similar behavior to corresponding radioligands reported in the literature [15, 18, 19, 21]. The results captured the typical features of each strategy design that impart to the targeting ligand and give hints for the design of FAP-targeting radio-therapeutics.

In conclusion, this head-to-head comparison indicated that dimerization of the FAPI small molecules and the cyclic peptide are two very promising strategies for enhancing tumor radiation dose, compared to FAPI monomers. In addition, the present study indicated that the therapeutic outcome of using albumin binders heavily depends on the selection of the albumin binding moiety. Considering the combination of tumor radiation dose (tumor uptake and residence), in vivo specificity, and tumor-to-background ratios (therapeutic index), the peptide showed certain advantages. However, the discrepancy of its performance between the different tumor models needs further investigation for concluding on any overall superiority compared to the other strategies and to FAPI small molecules.

## Supplementary Information

Below is the link to the electronic supplementary material.

Supplementary file1 (PDF 1.30 MB)

## Data Availability

The datasets generated during and/or analyzed during the current study are available from the corresponding author on reasonable request.
