# Supplementary material for: Head-to-head comparison of different classes of FAP radioligands designed to increase tumor residence time: monomer, dimer, albumin binders, and small molecules vs peptides
Source: Eur J Nucl Med Mol Imaging. 2023 Jun 1;50(10):3050–61. doi: 10.1007/s00259-023-06272-7 (PMC10382406; doi:10.1007/s00259-023-06272-7)
Supplement: Supplementary file 1 — Supplementary file1 (PDF 1.30 MB) [file 259_2023_6272_MOESM1_ESM.pdf]

Supplemental information

Head-to-head comparison of different classes of FAP radioligands designed to increase tumor residence time: monomer, dimer, albumin binders, and small molecules *vs* peptides

Millul Jacopo[1], Lennart Koepke[2], Gaonkar Raghuvir Haridas[1], Konstantin M.J. Sparrer[2], Rosalba Mansi[1] and Melpomeni Fani[1]

[1]Division of Radiopharmaceutical Chemistry, University Hospital Basel, Basel, Switzerland

[2]Institute of Molecular Virology, Ulm University Medical Center, Ulm, Germany

*Correspondence: melpomeni.fani@usb.ch

Table of contents

Abbreviations 3

General Remarks and Procedures 5

Synthesis of FAP-targeting ligands: FAPI-46, FAPI-46-F1, FAPI-46-F1D, FAPI-46-Ibu, FAPI-46-EB and FAP-2286 6

ESI/MS and HPLC profiles 8

Radiolabeling and radioHPLC chromatograms 11

Determination of lipophilicity (log *D)* 15

Transduction of HT-1080 and HEK-293 cells with lentiviral vectors harboring a hFAP expression cassette 16

hFAP expression in transduced HEK-293 and HT-1080 18

In vitro inhibition assay on hFAP 20

In vitro experiments in HT-1080.hFAP and HEK-293.hFAP 22

SPECT/CT imaging studies in tumor-bearing mice 24

Biodistribution results 26

Ex vivo blocking studies 32

In vitro autoradiography 34

Statistical analysis 36

Biodistribution in HT-1080.hFAP with 50 pmol – mass effect 40

Abbreviations

2-Cl-Trt	2-Cloro-Trityl
ACN	Acetonitrile
AUC	Area Under the Curve
BSA	Bovine Serum Albumin
Ca-DTPA	Calcium Diethylenetriamine pentaacetate
CAF	Cancer Associated Fibroblast
CT	Computed Tomography
DAP	Diamino-Proprionic Acid
DCM	Dichloromethane
DIPEA	N,N-Diisopropylethylamine
DMF	N,N’-Dimethylformamide
DOTA	1,4,7,10-Tetraazacyclododecane-1,4,7,10-tetraacetic acid
EDTA	Ethylenediaminetetraacetic Acid
FAP	Fibroblast Activation Protein α
FAPI	FAP Inhibitor
FBS	Fetal Bovine Serum
FDA	Food and Drug Administration
FMOC	9-Fluorenylmethoxycarbonyl
HATU	O-(7-azabenzotriazol-1-yl)-tetramethyl-uronium hexafluorophos-phate
hFAP	Human FAP
HPLC	High Performance Liquid Chromatography
Ibu	Ibuprofen (2-(4-isobutylphenyl)propanoic acid)
IC_{50}	Half maximal inhibitory concentration
I.A./g	Injected activity / gram

ivDde	1-(4,4-dimethyl-2,6-dioxocyclohex-1-ylidene)-3-methylbutyl
LC/MS	Liquid Chromatography / Mass Spectrometry
Lu	Lutetium
MEM	Minimum Essential Medium
MeOH	Methanol
PBS	Phosphate-Buffered Saline
PET	Positrom Emission Tomography
p.i.	Post Injection
PSMA	Prostate Specific Membrane Antigen
SPECT	Single Photon Emission Computed Tomography
TFA	Trifluoroacetic Acid
TFE	Trifluoro Ethane
TIPS	Triisopropylsilane
TRIS	Tris(hydroxymethyl)aminomethane
SD	Standard Deviation
w.t.	Wild Type

General Remarks and Procedures

All the solvents and reagents were purchased and used as supplied by Sigma Aldrich (Switzerland), AstaTech (USA) and VWR International (Switzerland) in HPLC or analytical grade. FAPI-46-F1-EB was purchased from WuXi AppTech (China).

Liquid chromatography mass spectrometry (LC/MS) was run on a LCMS-2020 Shimadzu system equipped with a Gemini C-6 Phenyl column (4.6 x 250 mm, 5 µm particle size). The gradient used was 15-65% solvent *B* in 15 min (*A* = H_2O [0.1%TFA], *B* = ACN [0.1% TFA]) at a flow rate of 1.0 mL/min. Radio-HPLC was performed on a Shimadzu instrument (Shimadzu Corporation, Kyoto, Japan) connected to a GABI radioactivity-HPLC-flow-monitor γ-spectrometer (Elysia-raytest, Straubenhardt, Germany). Radioligands were analyzed using Phenomenex Jupiter Proteo C12 (90 Å, 250 × 4.6 mm) column using the gradient 15-65 % *B* in 15 min (*A* = H_2O [0.1%TFA], *B* = ACN [0.1% TFA]) with a flow rate of 1 mL/min. Quantitative γ-counting was carried out on a Cobra 5003 γ-system well counter from Packard Instruments (Meriden, CT, USA).

Synthesis of FAP-targeting ligands: FAPI-46, FAPI-46-F1, FAPI-46-F1D, FAPI-46-Ibu, FAPI-46-EB and FAP-2286

FAPI-46 was synthesized following published procedures as mentioned in the manuscript. FAPI-46-F1 was synthesized by coupling the FAP-binding moiety (S)-N-(2-(2-cyano-4,4-difluoropyrrolidin-1-yl)-2-oxoethyl)-6-(methyl(3-(piperazin-1-yl)propyl)amino)quinoline-4-carboxamide to asparagine, while the N-termini was used for DOTA conjugation.

Aspartic acid was used as a linker for the synthesis of FAPI-46-F1D and FAPI-46-EB. In the dimer FAPI-46-F1D, two FAP-binding moieties were coupled to the carboxylic acids of the aspartic acid, while FAPI-46-EB was synthesized by adding the Evan's Blue moiety in the δ-carboxylic acid of the linker. For both molecules, N-termini was conjugated to DOTA.

The peptidic sequence of FAP-2286 was synthesized with the automatic synthesizer Liberty Blue by microwave-assisted synthesis, starting from Cys-Cl-Trityl resin. After cleavage of the peptide, 1,3,5-tris(bromomethyl)benzene was used to cyclize the molecule, which was further functionalized with cysteamine. The N-termini was used for DOTA conjugation.

The synthesis of FAPI-46-Ibu was performed as follow:

50 mg of 2-Cl-trityl resin were soaked in 5 mL of dry DCM for 15 minutes. The Fmoc protected amino acid Fmoc-DAP(ivDde)-OH (0.09 mmol, 50 mg) and DIPEA (4 eq.) were dissolved in 3 mL of dry DCM. The mixture was added to the resin and stirred for 120 min. The resin was then washed 3 times with a mixture of DCM/MeOH/DIPEA (17:2:1), then 3 times with DCM, 2 times with DMF and 2 times with DCM. To evaluate the yield of the reaction, a 20% solution of Piperidine in DMF was used to deprotect the Fmoc protecting group, and its concentration was evaluated with a spectrophotometer, using an ε=7800. 0.076 mmol were obtained.

DOTA-3tBu (2 eq, 0.15 mmol, 88 mg), HATU (2 eq, 0.15 mmol, 55 mg), DIPEA (4 eq, 0.3 mmol, 60 µL) were dissolved in DMF and let react for 1h at room temperature, under vigorous

agitation. Keiser test was performed to confirm the coupling. ivDde group was removed by treating the resin 10 times with 5% hydrazine in DMF for 3 minutes. The fractions were checked at the spectrophotometer until the signal at 290 nm was completely disappeared. Ibuprofen (2 eq, 0.15 mmol, 31 mg), HATU (2 eq, 0.15 mmol, 55 mg), DIPEA (4 eq, 0.3 mmol, 60 µL) were dissolved in DMF and let react for 1h at room temperature, under vigorous agitation. Keiser test was performed to confirm the coupling. The peptide was cleaved from the resin with DCM/TFE (8:2) for 1h, 3 times. The fractions were collected, the resin washed with DCM and then dried. Ether was added to precipitate the peptide, which was collected and used as such.

The crude peptide (1.2 eq, 0.048 mmol, 40 mg), HATU (1.2 eq, 0.04 mmol, 55 mg), and the FAP-binding moiety (*S*)-N-(2-(2-cyano-4,4-difluoropyrrolidin-1-yl)-2-oxoethyl)-6-(methyl(3-(piperazin-1-yl)propyl)amino)quinoline-4-carboxamide (1 eq, 0.04 mmol, 20 mg) were dissolved in 500 µL of DCM. DIPEA (4 eq, 0.08 mmol, 14µl) was added dropwise and the reaction was mixed at room temperature and followed via LC/MS. After completion, 60 µL of TIPS and 700 µL of TFA were added and the reaction was stirred 48h. The solution was precipitated in cold ether, centrifuged, and the pellet was dissolved in water and directly purified via HPLC (Method: 10 to 60% *B* in 10 min) to obtain a red powder (16 mg, 0.0138 mmol, 35% yield).

ESI/MS and HPLC profiles

FAPI-46

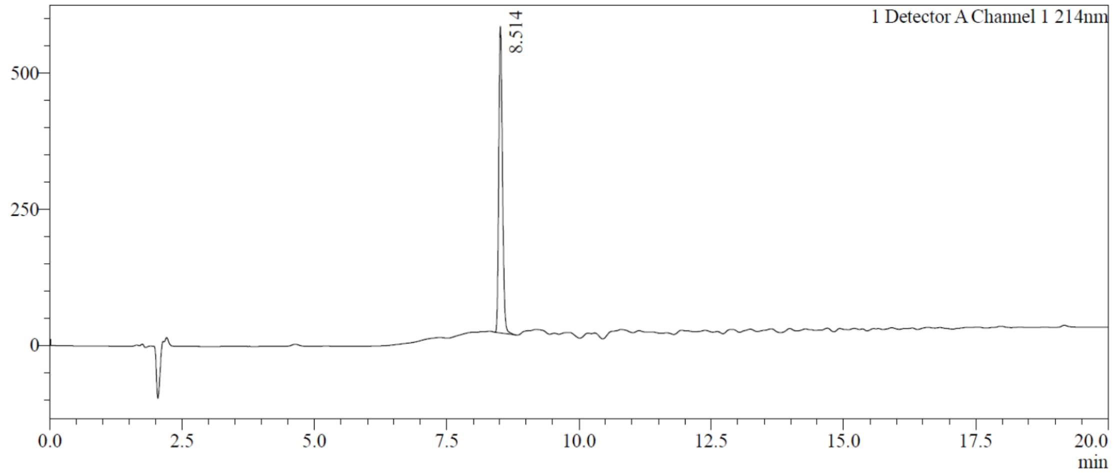

Method: 10-65% *B* in15 min

MS (ESI) m/z calculated for $[C_{41}H_{57}F_2N_{11}O_9]^+$: 886.43 $[M+H]^+$, found: 886.55.

Purity: 99%

FAPI-46-F1

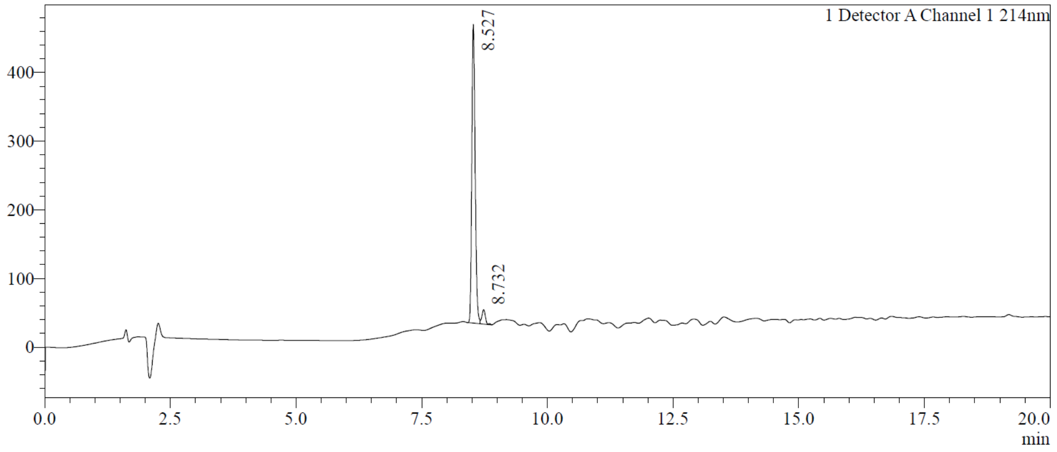

Method: 10-60% *B* in15 min

MS (ESI) m/z calculated for $[C_{45}H_{63}F_2N_{13}O_{11}]^+$: 1000.08 $[M+H]^+$, found: 1000.50.

Purity: 93%

FAPI-46-F1D

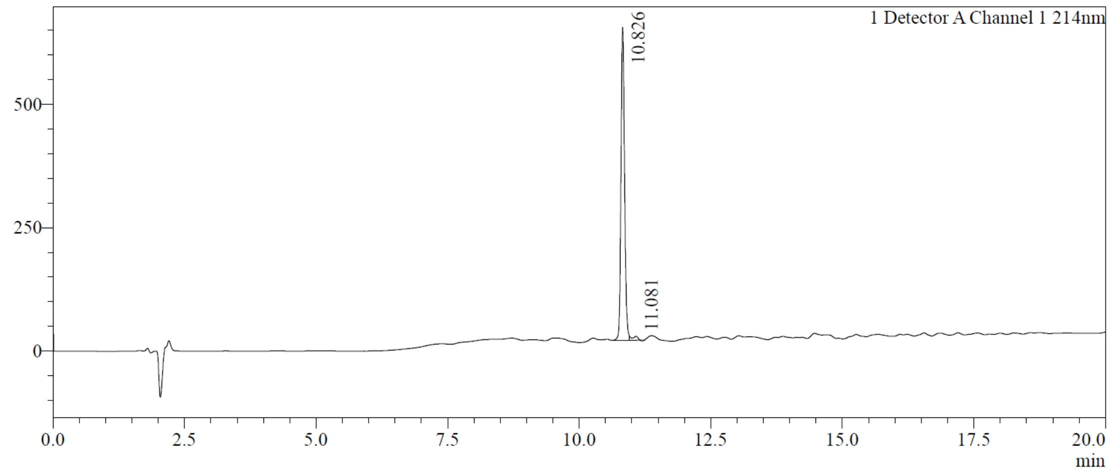

Method: 10-60% *B* in15 min

MS (ESI) m/z calculated for $[C_{70}H_{91}F_4N_{19}O_{13}]^+$: 1482.61 $[M+H]^+$, found: 1484.70.

Purity: 98.5%

FAPI-46-Ibu

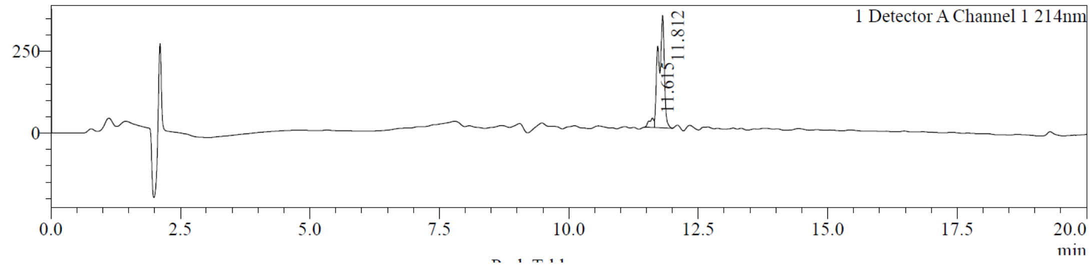

Method: 15-80% *B* in15 min

MS (ESI) m/z calculated for $[C_{57}H_{79}F_2N_{13}O_{11}]^+$: 1160.34 $[M+H]^+$, found: 1162.0.

The presence of a double peak is due to the fact that Ibuprofen was purchased and used as racemate, generating two diastereoisomers.

Purity: 93%

FAPI-46-EB

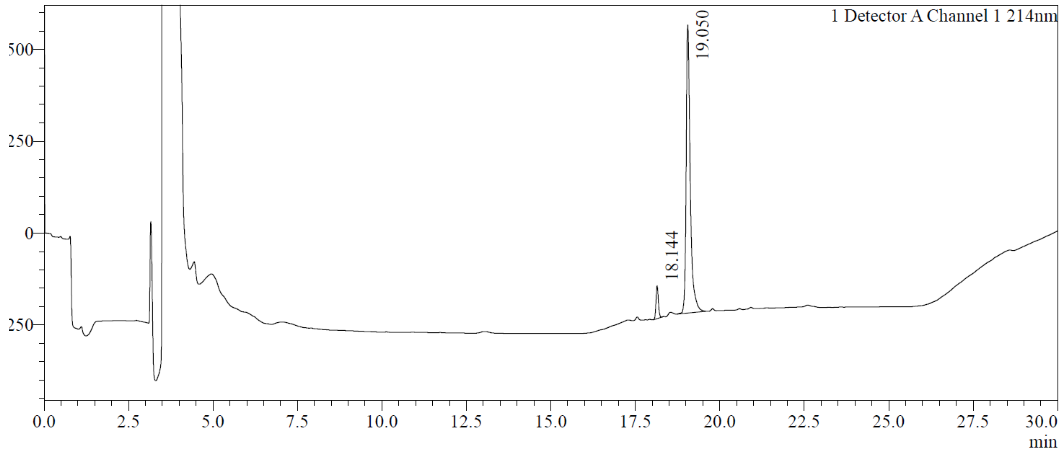

Method: 30-45% *B* in 30 min

MS (ESI) m/z calculated for $[C_{69}H_{82}F_2N_{16}O_{18}S_2]^+$: 1524.54 $[M+H]^+$, found: 1526.54

Purity: 92%

FAP-2286

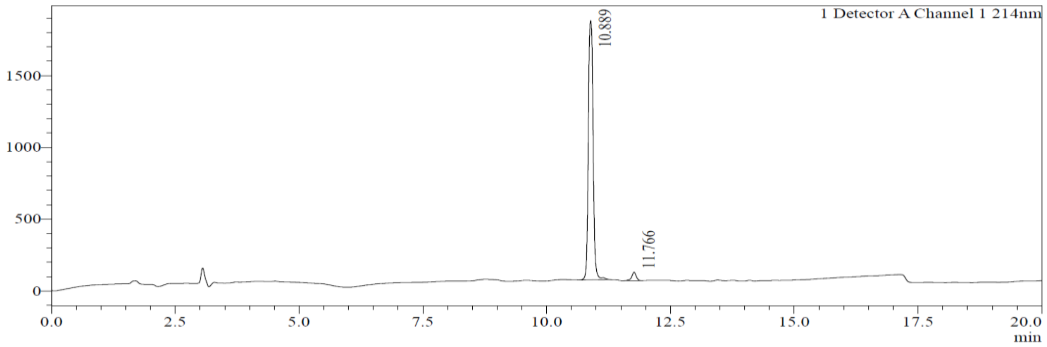

Method: 15-80% *B* in15 min

MS (ESI) m/z calculated for $[C_{67}H_{99}N_{13}O_{18}S_3]^+$: 1470.78 $[M+H]^+$, found: 1470.70

Purity: 93%

Radiolabeling and radioHPLC chromatograms

All the ligands were labeled with Lu-177 following well-established procedures. An apparent molar activity of 8-10 MBq/nmol was used for biodistribution experiments, while an apparent molar activity of 24-36 MBq/nmol was used for SPECT/CT imaging and *in vitro* experiments. All radiochromatograms were recorded using the method 10% to 65% *B* in 15 min. **Supplemental Table 1** describes the conditions and retention time of all radioligands. Representative radioHPLC chromatograms are provided below.

Supplemental Table 1. Radiolabeling conditions and retention time of all studied radioligands.

Radioligand	Radiolabeling conditions	Retention time (min)
**[^{177}Lu]Lu-FAPI-46**	Ascorbate buffer, 0.2M, pH=4, 95°C, 25 min	8.2±0.2
**[^{177}Lu]Lu-FAPI-46-F1**	Ascorbate buffer, 0.2M, pH=4, 95°C, 25 min	7.7±0.2
**[^{177}Lu]Lu-FAPI-46-F1D**	Ascorbate buffer, 0.2M + 10% EtOH, pH=4, 95°C, 25 min	9.5±0.2
**[^{177}Lu]Lu-FAPI-46-Ibu**	Ascorbate buffer, 0.2M, pH=4, 95°C, 25 minutes	13.1±0.2
**[^{177}Lu]Lu-FAPI-46-EB**	Ammonium acetate buffer, 0.4M, pH=5, 95°C, 25 min	12.1±0.2
**[^{177}Lu]Lu-FAP-2286**	Ammonium acetate buffer, 0.4M + 10% EtOH, pH=5, 95°C, 25 min	13.4±0.2

[^{177}Lu]Lu-FAPI-46

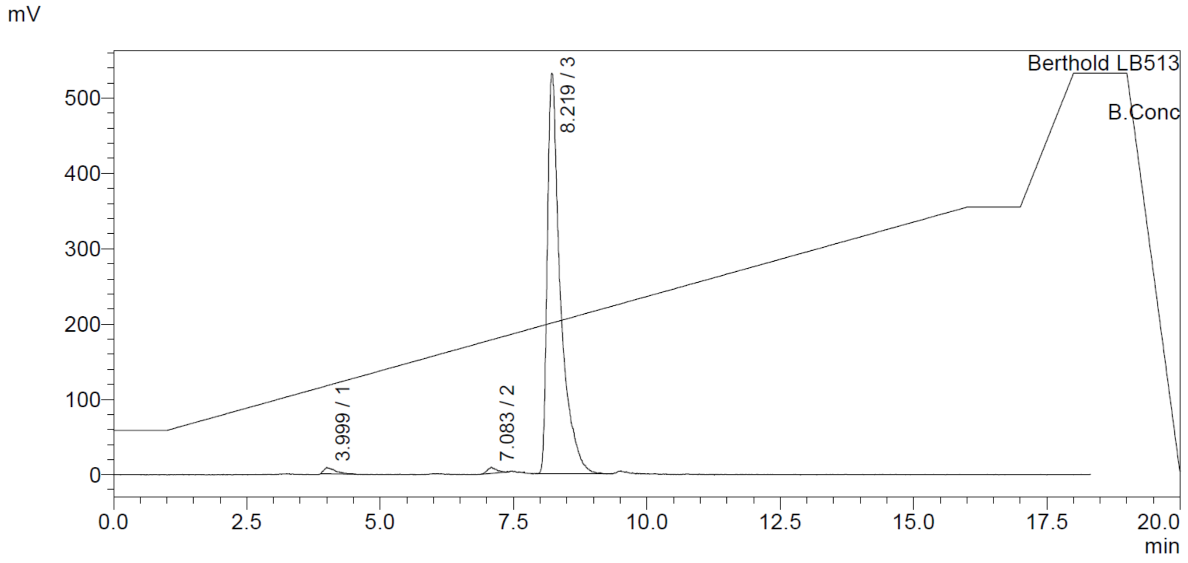

Radiochemical purity: ≥95%

[^{177}Lu]Lu-FAPI-46-F1

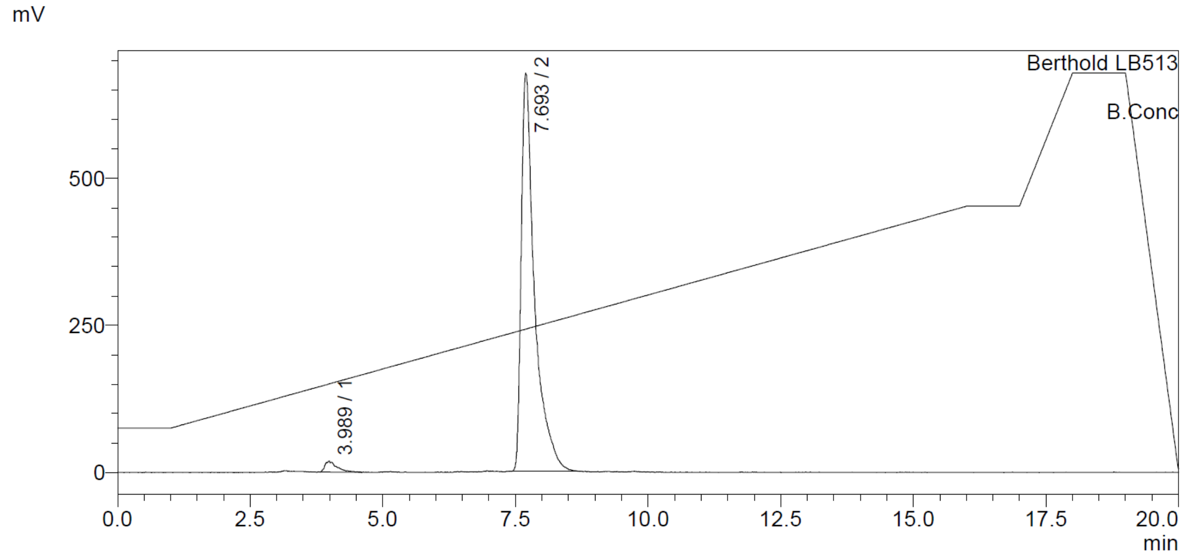

Radiochemical purity: ≥95%

[^{177}Lu]Lu-FAPI-46-F1D

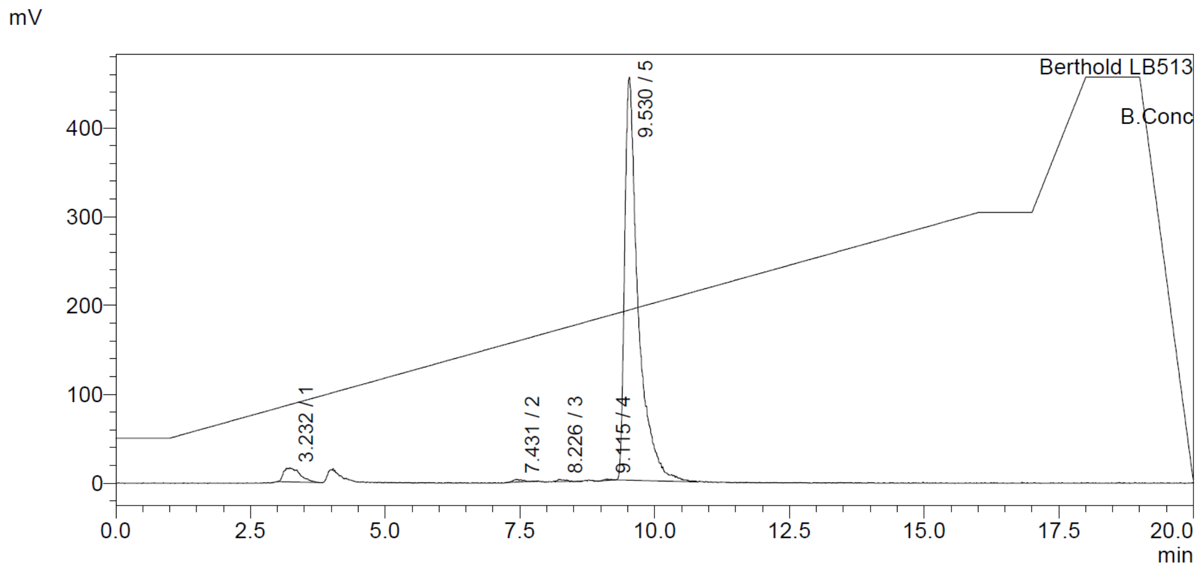

Radiochemical purity: ≥95%

[^{177}Lu]Lu-FAPI-46-Ibu

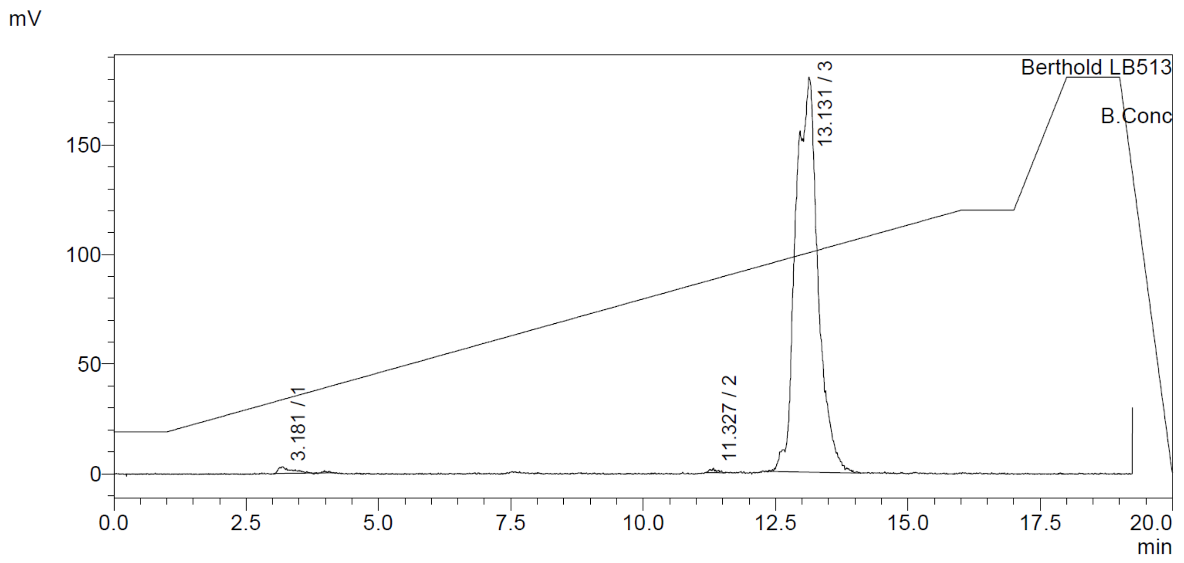

Radiochemical purity: ≥95%

[^{177}Lu]Lu-FAPI-46-EB

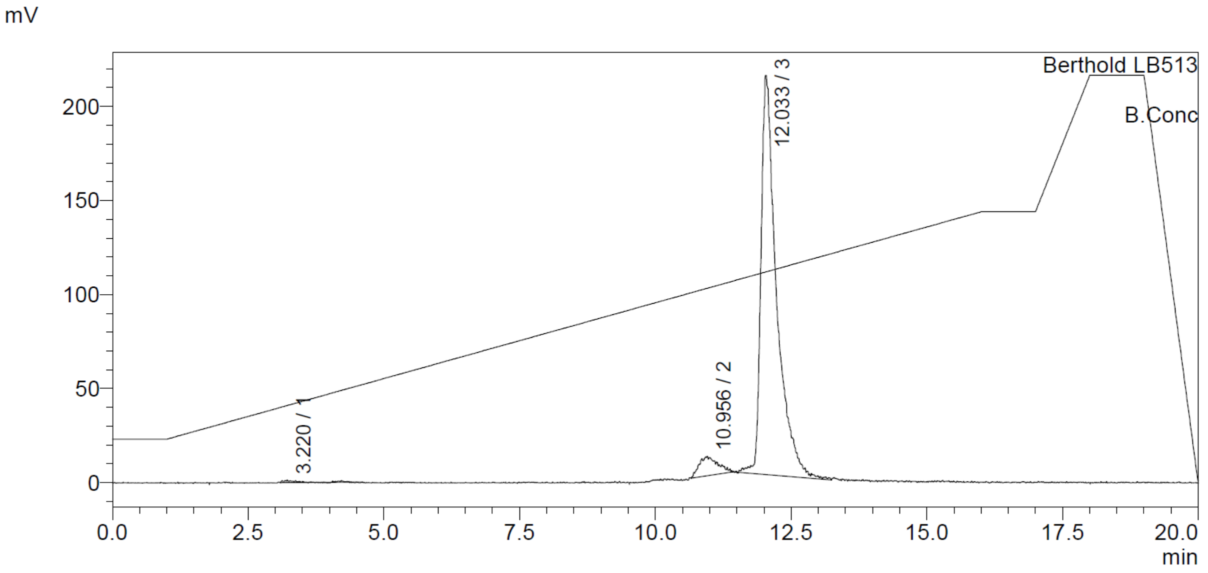

Radiochemical purity: ≥94%

[^{177}Lu]Lu-FAP-2286

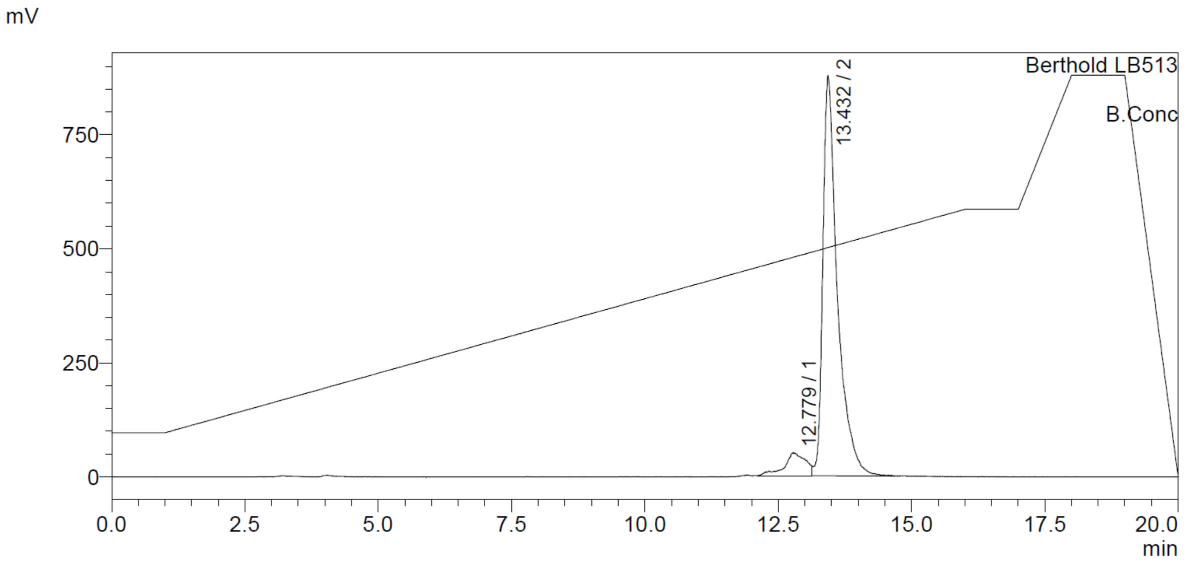

Radiochemical purity: ≥92%

Determination of lipophilicity (log *D*)

The distribution coefficient (log *D*) of the radioligands was determined by the shake-flask method. In a pre-lubricated Eppendorf tube, a pre-saturated mixture of 500 µL of 1-octanol and 500 µL of PBS (phosphate-buffered saline) at pH 7.4 was added. An aliquot of 10 pmol in 10 µL of the radioligand was added to this mixture, shaken for 30 min, and then centrifuged at 3000 rcf for 10 min to achieve phase separation. Aliquots of 100 µL were removed from the 1-octanol and from the PBS phases, and the activity was measured in a γ-counter. The distribution coefficient was calculated as the average log ratio value of the radioactivity in the organic fraction and PBS fraction.

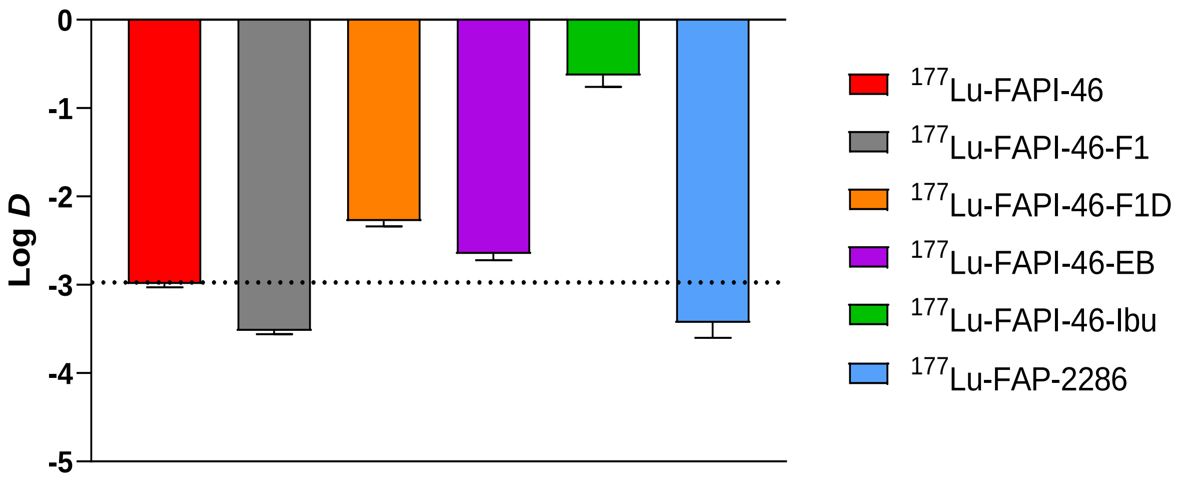

Supplemental Figure 1. Log $D_{(pH=7.4)}$ of all the radioligands in comparison with [^{177}Lu]Lu-FAPI-46 (dotted line). All the radioligands, but [^{177}Lu]Lu-FAPI-46-Ibu, exhibited a high hydrophilic character which was comparable with the one of [^{177}Lu]Lu-FAPI-46. The Ibuprofen-conjugate was found to be the most lipophilic radioligand among the series.

Transduction of HT-1080 and HEK-293 cells with lentiviral vectors harboring a hFAP expression cassette

PEI mediated transfection was carried out by suspending plasmid DNA in 250 µL Opti-MEM per well and in parallel, suspending PEI (2 µg PEI per 1 µg plasmid DNA, stock solution 1 µg/µL) in 250 µL Opti-MEM for each well. Both suspensions were mixed 1:1. After incubation at RT for 20 min, 500 µL of transfection mix was added to each well carefully without disturbing the monolayer.

A cDNA clone of FAP in a pcDNA3.1 vector was purchased from GenScript (OHu27944D). pCSC-SP-PW-GFP (aka: pBOB-GFP, 12337; RRID:Addgene_12337) was a gift from Inder Verma (Addgene, #12337). The ORF of FAP was subcloned into the pBOB-GFP vector replacing the GFP ORF using Gibson assembly. To this end, the insert was amplified by PCR using the Phusion High-Fidelity PCR Kit according to the manufacturer's instructions with the primers FAP-f and FAP-r purchased from Biomers. The pBOB-GFP vector was linearized by digestion with the BamHI and PmeI restriction enzymes. The construct (pBOB-FAP) was assembled from the insert and the linearized vector using the HiFi DNA Assembly Cloning Kit (NEB E5520S) as per instructions.

The GFP ORF was removed from pBOB-GFP to produce an empty control using Gibson assembly. To this end, the insert was ordered from Biomers. The pBOB-GFP vector was linearized by digestion with the BamHI and PmeI restriction enzymes. The construct (pBOB-empty) was assembled from the insert and the linearized vector using the HiFi DNA Assembly Cloning Kit (NEB E5520S) as per instructions.

To produce lentiviral vectors, HEK293T cells were seeded in 6-well plates in 2 mL Dulbecco's Modified Eagle Medium supplemented with 10 % (v/v) fetal bovine serum, 100 U/mL penicillin, 100 µg/mL streptomycin and 2 mM L-glutamine (referred to as DMEM3x) using 900,000 cells per well and cultured overnight. The cells were transfected with pBOB-FAP or

pBOB-empty (1.75 µg per well) and the three helper plasmids pMDLg (0.87 µg per well, 12251: RRID:Addgene_12251), pRSV-Rev (0.44 µg per well, 12253; RRID:Addgene_12253), pMD2.G (0.5 µg per well, 12259; RRID:Addgene_12259) using PEI transfection as described in the section above. 4-6 h after the transfection, the medium was replaced with 2 mL DMEM3x. 48 h after the transfection, the lentiviral particles were harvested by taking the supernatant, pelleting the cell debris by centrifugation (2,500 g for 3 min) and filtering the stocks through 0.45 µm filters. The cleared lentiviral stocks were stored at -80°C.

The target cell lines (e.g. HEK293 or HT-1080) were seeded in 6-well plates in 1.7 mL medium using 1 million cells per well. 300 µL lentiviral stock (hFAP or control) was added to the target cells and they were cultured for 72 h. The transduced cells were washed with PBS and detached using trypsin (37°C for 5 min). The cells were then washed again with PBS. Next, the cells were suspended in 1 mL PBS and sorted by fluorescence-activated cell sorting (FACS) into 96-well F-bottom plates containing 200 µL DMEM3x targeting 1 cell per well. The single cells were expanded into clonal cell lines. Expression of hFAP on the cell surface was verified by antibody staining (Human Fibroblast Activation Protein alpha /FAP PE-conjugated Antibody R&D Systems FAB3715P-025) and analysis in flow cytometry before cryo-stocks were prepared and stored at -80°C.

<u>hFAP expression in transduced HEK-293 and HT-1080</u>

HEK-293 ctrl C1, HEK-293.hFAP D5, HT-1080 ctrl, and HT-1080.hFAP cells were seeded in flat-bottom 96-well plates at 50,000 cells in 200 µL DMEM3x per well. The cells were cultured overnight, before they were detached using trypsin and transferred to a 96-well v-bottom plate. The cells were washed once with PBS supplemented with 1% FBS and incubated with the hFAP-antibody (R&D Systems, FAB3715P-025) or the isotype control (R&D Systems, IC002P) for 30 min at 4°C. After the incubation, the cells were washed once with PBS supplemented with 1% FBS and then fixed using in 4% PFA in PBS for 30 min at RT. After the fixation, the supernatant was removed and the cells were suspended in PBS supplemented with 1% FBS. The cells were analyzed in flow cytometry using a CytoFLEX LX, measuring the PE fluorescence intensity.

A comparison between the relative expression level of FAP in the two cell lines used is reported in **Supplemental Figure 2**.

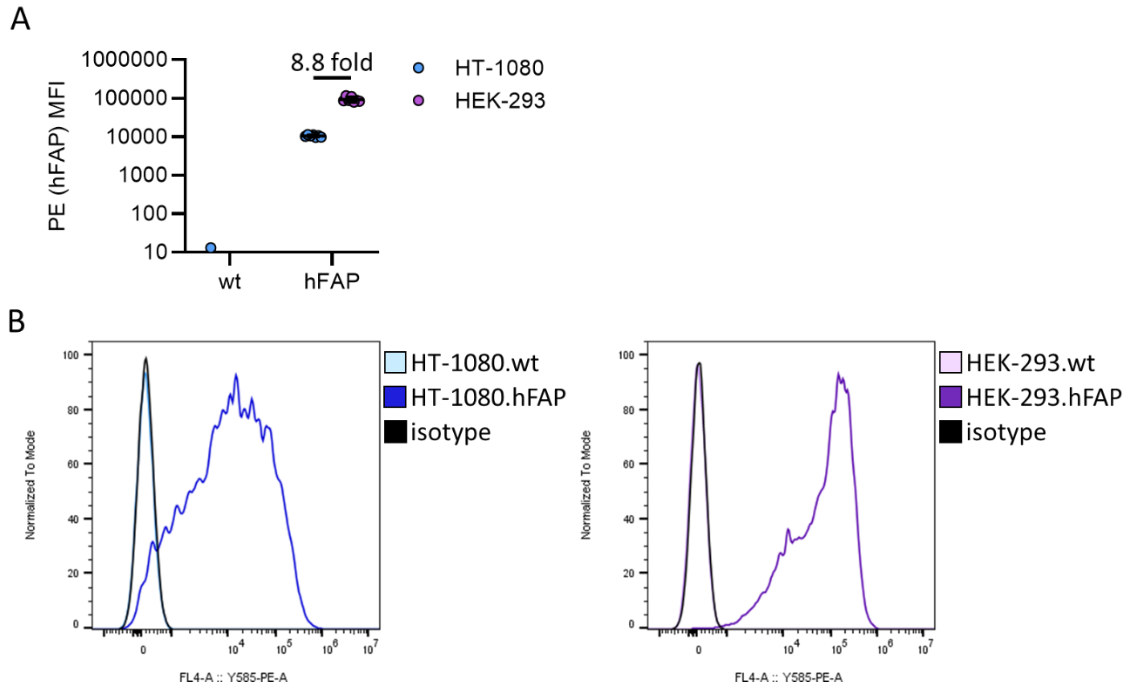

Supplemental Figure 2. A) Absolute median fluorescence intensity (MFI) of HT-1080 and HEK-293 cells (hFAP and wt) stained with an PE-labelled anti-FAP antibody, measured in flow

cytometry. MFI of the isotype control was subtracted to remove the background. Data are shown as mean±SD, n=7. **B)** Representative histograms depicting the fluorescence intensity of HT-1080 (left) and HEK-293 (right) cells (hFAP and wt) stained with an PE-labelled anti-FAP antibody or an PE-labelled isotype control.

***In vitro* inhibition assay on hFAP**

The enzymatic activity of hFAP on the substrate Z-Gly-Pro-AMC was measured at room temperature on a microtiter plate reader, monitoring the fluorescence at an excitation wavelength of 360 nm and an emission wavelength of 465 nm. The assay was performed by mixing the substrate (20 µM), hFAP (200 pM, constant), and the ligands in assay buffer (50 mM Tris, 1 M NaCl, 1 mg/mL BSA, pH=7.5), with serial dilution of the inhibitors ranging from 250 nM to 2 fM, 1:2 in a total volume of 20 µL.

Experiments were performed in triplicate, and the mean fluorescence values were fitted using GraphPad Prism 9 (equation used: Y = Bottom + (Top − Bottom)/(1 + ((X^HillSlope)/(IC50^HillSlope)))). The IC_{50} value is defined as the concentration of ligand (inhibitor) required to reduce the enzyme activity by 50% after the addition of the substrate. The results are presented in the **Supplemental Figure 3**.

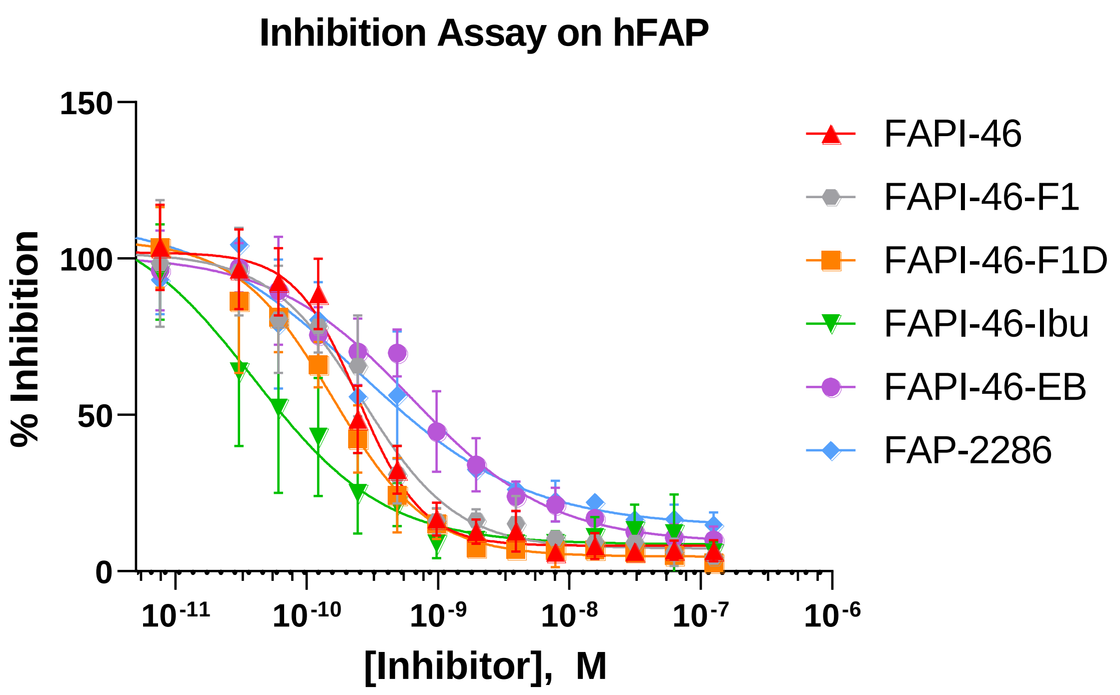

Supplemental Figure 3: Inhibition assay performed on hFAP showed excellent inhibition properties from all the derivatives. FAPI-46-F1D and FAPI-46-Ibu ((IC_{50} = 157.8±14.5 and

39.4±16.1 pM, respectively) showed enhanced inhibition properties compared to FAPI-46 (IC_{50} = 247.0±17 pM). FAPI-46-F1 and FAP-2286 presented very similar IC_{50} compared to FAPI-46, while FAPI-46-EB showed the lowest inhibition activity against hFAP (IC_{50} = 265.6±35.9, 634.3±102.3, and 247.6±71.1 pM for FAPI-46-F1, FAPI-46-EB and FAP-2286, respectively).

In vitro experiments in HT-1080.hFAP and HEK-293.hFAP

Cellular uptake

HT-1080.hFAP, HT-1080.wt, HEK-293.hFAP and HEK-293.wt cells were seeded in a 24-well plate at a concentration of 1.8×10^5 cells/well in 400 µL of culture medium 24 h before the experiment. The cells were then preconditioned in 360 µL of assay medium (MEM medium without supplements) at 37°C for 60 min. 40 µL of a 2 nM solution of ^{177}Lu-labeled radioligand was added and the cells were incubated at 37°C. Cellular uptake was interrupted at different time points (15 min, 1 hour and 4 hours), by washing twice with ice-cold PBS. Cell surface-bound radioligand was obtained by washing cells twice with ice-cold glycine buffer (pH 2.8), followed by a collection of the internalized fraction with 1 M NaOH. The activity in each fraction was measured in a γ-counter (Cobra II). The results were expressed as the mean ± standard deviation of the percentage of the applied radioactivity, after subtracting the non-specific uptake in the HT-1080.wt cells and HEK-293.wt cells, respectively. Experiments were performed in duplicate (n=2).

The results are presented in **Supplemental Figure 4 A-B and Supplemental Table 2**.

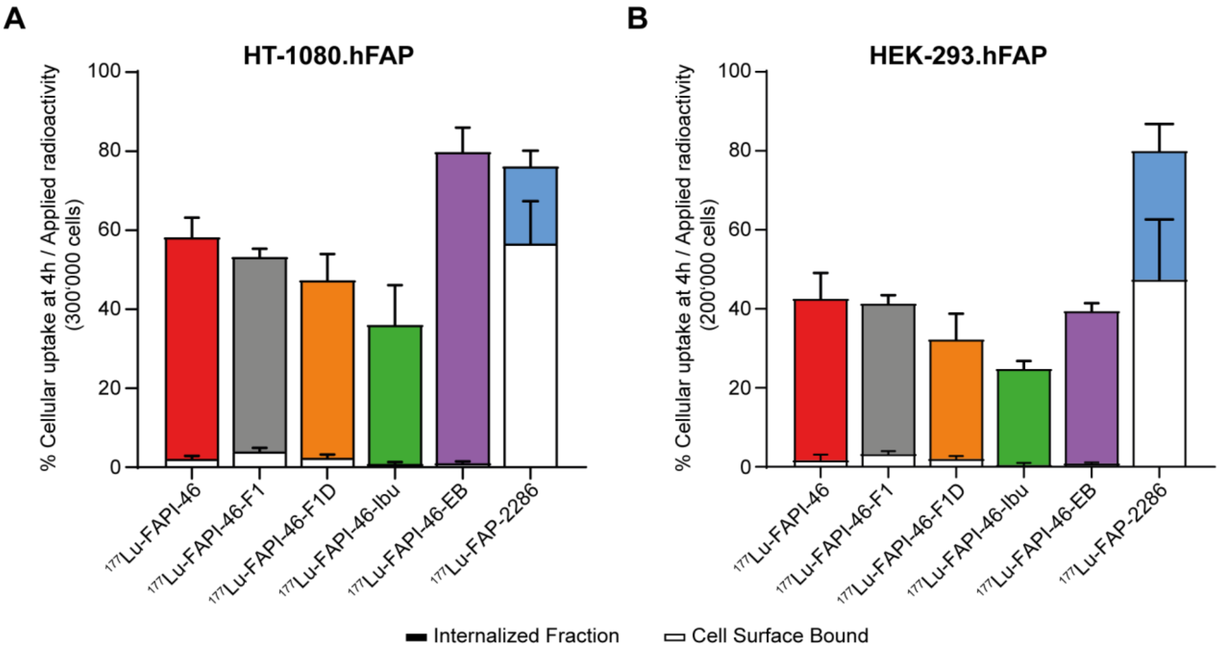

Supplemental Figure 4. Mean ± standard deviation of the specific cellular uptake of the radioligands in HT-1080.hFAP (**A**) and HEK-293.hFAP (**B**) after 4h at 37°C. All radioligands showed selective uptake in FAP-positive cells, with high internalization fraction. Only FAP-2286 was found to be mainly bound to the cell surface.

Supplemental Table 2. Cellular distribution (expressed as % of Cellular Surface Bound and Internalized Fraction of the applied radioactivity) in HT-1080.hFAP and HEK-293.hFAP cells.

**Radioligand**	**Cell Surface Bound Fraction**			**Internalized Fraction**		
	15 min	1 h	4 h	15 min	1 h	4 h
**HT-1080.hFAP cells**						
**[^{177}Lu]Lu-FAPI-46**	1.6±0.6	2.1±0.9	2.5±1.1	36.0±2.6	52.4±4.8	56.1±5.0
**[^{177}Lu]Lu-FAPI-46-F1**	2.4±0.2	2.6±0.5	4.0±0.9	33.1±0.8	42.9±3.9	49.3±2.1
**[^{177}Lu]Lu-FAPI-46-F1D**	1.6±0.2	2.3±0.2	2.4±0.8	32.7±2.2	39.3±3.2	44.9±6.7
**[^{177}Lu]Lu-FAPI-46-Ibu**	0.5±0.2	1.2±0.3	0.8±0.5	20.0±3.2	34.6±6.3	35.2±10.1
**[^{177}Lu]Lu-FAPI-46-EB**	0.1±0.1	1.5±0.7	1.0±0.5	15.0±4.6	41.7±14.2	78.8±6.2
**[^{177}Lu]Lu-FAP-2286**	37.5±4.3	50.3±8.5	56.6±10.7	4.9±0.8	12.2±1.2	19.5±4.0
**HEK-293.hFAP cells**						
**[^{177}Lu]Lu-FAPI-46**	1.6±1.1	2.3±1.7	1.7±1.4	26.5±6.6	35.8±5.3	40.9±6.5
**[^{177}Lu]Lu-FAPI-46-F1**	2.2±0.1	2.8±0.3	3.3±0.7	21.3±0.8	32.9±3.5	38.0±2.1
**[^{177}Lu]Lu-FAPI-46-F1D**	1.3±0.6	1.6±0.8	2.1±0.7	25.2±3.2	24.8±3.7	30.2±6.5
**[^{177}Lu]Lu-FAPI-46-Ibu**	0.6±0.1	0.4±0.2	0.5±0.5	15.4±2.1	18.1±3.2	24.3±2.0
**[^{177}Lu]Lu-FAPI-46-EB**	0.8±0.2	1.0±0.6	0.9±0.2	23.0±7.7	21.5±5.8	38.5±2.0
**[^{177}Lu]Lu-FAP-2286**	28.1±5.2	38.9±10.7	47.7±15.2	8.4±3.2	18.9±5.8	32.6±6.8

SPECT/CT imaging studies in tumor-bearing mice

Topograms and helical CT scans were performed using the following parameters: X-ray tube current: 177 mA, X-ray tube voltage 45 kVp, 90 seconds and 180 frames per rotation, pitch 1. A helical SPECT scan was acquired using multipurpose pinhole collimators (APT1), 20% energy window width centered symmetrically over the 208 and 113 keV g-peaks of ^{177}Lu, 24 projections, and 1200 s per projection.

SPECT images were reconstructed iteratively and filtered using the HiSPECT software package (version 1.4.1876, SciVis GmbH, Goettingen, Germany) and the manufacturer's algorithm (3 subsets, 9 iterations, 35% post-filtering, 128x128 matrix, zoom 1, 30x20 mm transaxial field of view, resulting in a pixel size of 0.3 mm). CT images were reconstructed using CTReco (version r1.146), with a standard filtered back projection algorithm (exact cone beam) and post-filtered (RamLak, 100% frequency cut-off), resulting in a pixel size of 0.2 mm. Co-registered images were visualized in the three orthogonal planes using maximum intensity projection with InVivoScope (version 1.43, Bioscan Inc.)

Supplemental Table 3. Percentage of the *post mortmem* remaining activity in HT-1080 and HEK-293 bearing mice 4h p.i.. The data are absolute quantification values obtained by dose-calibrator and decay-corrected.

**Radioligand**	% remaining activity in HT-1080 xenografts	% remaining activity in HEK-293 xenografts
**[^{177}Lu]Lu-FAPI-46**	18	38
**[^{177}Lu]Lu-FAPI-46-F1**	26	n.d.
**[^{177}Lu]Lu-FAPI-46-F1D**	35	43
**[^{177}Lu]Lu-FAPI-46-Ibu**	47	57
**[^{177}Lu]Lu-FAPI-46-EB**	90	78
**[^{177}Lu]Lu-FAP-2286**	8	6

n.d. not done

The injected activity and *post-mortem* activity measured 4h p.i. of the radioligands indicated highest body retain for [^{177}Lu]Lu-FAPI-46-EB and highest washout for [^{177}Lu]Lu-FAP-2286, among all radioligands.

Biodistribution results

Supplemental Table 4. Biodistribution studies of [^{177}Lu]Lu-FAPI-46 in HT1080.hFAP and HEK-293.hFAP and the corresponding wild type dual xenografts at 4h, 24h, 72h and 120h p.i. Results are expressed as mean of the % injected activity per gram of tissue (%I.A./g) ± standard deviation (SD).

**Organ**	**HT-1080 xenografts**				**HEK-293 xenografts**		
	**4h (n=4)**	**24h (n=4)**	**72h (n=4)**	**120h (n=4)**	**4h (n=5)**	**24h (n=4)**	**72h (n=4)**
Blood	0.43±0.04	0.03±0.01	0.00±0.00	0.00±0.00	0.65±0.08	0.03±0.01	0.00±0.00
Heart	0.21±0.01	0.04±0.01	0.02±0.01	0.01±0.00	0.30±0.04	0.04±0.02	0.01±0.00
Lung	0.29±0.03	0.05±0.02	0.03±0.00	0.01±0.00	0.41±0.08	0.04±0.01	0.01±0.00
Liver	0.29±0.02	0.21±0.06	0.24±0.05	0.12±0.06	0.35±0.05	0.19±0.07	0.10±0.04
Pancreas	0.42±0.04	0.06±0.01	0.03±0.01	0.01±0.00	0.63±0.11	0.05±0.02	0.01±0.00
Spleen	0.19±0.01	0.08±0.02	0.09±0.02	0.04±0.01	0.22±0.05	0.07±0.02	0.03±0.01
Stomach	0.20±0.01	0.04±0.01	0.05±0.02	0.01±0.00	0.36±0.05	0.03±0.02	0.01±0.00
Intestine	0.19±0.03	0.05±0.01	0.02±0.00	0.01±0.00	0.30±0.06	0.03±0.01	0.01±0.00
Adrenal	0.48±0.07	0.12±0.04	0.08±0.02	0.03±0.02	0.58±0.18	0.09±0.06	0.03±0.02
Kidney	1.19±0.34	0.59±0.05	0.39±0.12	0.14±0.04	1.33±0.19	0.38±0.06	0.15±0.10
Muscle	0.58±0.38	0.09±0.05	0.04±0.03	0.01±0.01	0.49±0.14	0.08±0.03	0.01±0.00
Femur	2.52±0.38	0.61±0.14	0.73±0.08	0.25±0.15	2.22±0.58	0.55±0.22	0.19±0.06
hFAP-tumor	9.63±1.79	2.68±0.74	1.58±0.37	0.40±0.13	10.34±4.55	3.39±1.65	0.44±0.11
wt-tumor	1.02±0.18	0.35±0.13	0.17±0.04	0.04±0.01	1.40±0.39	0.28±0.07	0.05±0.01

Supplemental Table 5. Biodistribution studies of [^{177}Lu]Lu-FAPI-46-F1D in HT1080.hFAP and HEK-293.hFAP and the corresponding wild type dual xenografts at 4h, 24h, 72h and 120h p.i. Results are expressed as mean of the % injected activity per gram of tissue (%I.A./g) ± standard deviation (SD).

**Organ**	**HT-1080 xenografts**				**HEK-293 xenografts**		
	**4h (n=4)**	**24h (n=4)**	**72h (n=4)**	**120h (n=4)**	**4h (n=6)**	**24h (n=4)**	**72h (n=4)**
Blood	1.21±0.22	0.60±0.05	0.04±0.01	0.01±0.00	1.51±0.28	0.59±0.08	0.05±0.01
Heart	0.56±0.07	0.50±0.05	0.27±0.06	0.27±0.07	0.66±0.10	0.50±0.07	0.30±0.04
Lung	0.87±0.21	0.53±0.01	0.18±0.03	0.15±0.02	0.93±0.13	0.55±0.07	0.25±0.04
Liver	0.67±0.16	1.37±0.04	1.23±0.27	1.62±0.15	0.83±0.25	1.35±0.15	1.66±0.31
Pancreas	1.10±0.17	1.04±0.12	0.31±0.08	0.31±0.03	1.14±0.27	0.72±0.11	0.33±0.03
Spleen	0.47±0.08	0.72±0.01	0.88±0.26	1.02±0.18	0.49±0.07	0.72±0.10	0.93±0.10
Stomach	0.51±0.10	0.44±0.04	0.24±0.03	0.21±0.03	0.58±0.14	0.47±0.03	0.27±0.02
Intestine	0.57±0.17	0.43±0.12	0.19±0.07	0.18±0.05	0.51±0.16	0.45±0.14	0.25±0.05
Adrenal	1.45±0.35	1.54±0.04	0.83±0.48	0.81±0.09	1.24±0.28	0.87±0.26	1.05±0.21
Kidney	1.25±0.08	1.02±0.16	0.87±0.20	0.78±0.23	1.28±0.19	0.94±0.06	0.73±0.06
Muscle	1.35±0.43	0.88±0.10	0.41±0.21	0.32±0.09	1.05±0.27	0.80±0.11	0.41±0.06
Femur	4.91±0.75	2.89±0.08	2.02±0.32	1.78±0.24	5.19±0.86	3.04±0.27	2.09±0.23
hFAP-tumor	10.47±2.47	6.42±0.89	3.57±0.73	1.57±0.30	17.16±4.63	5.89±1.29	1.43±0.20
wt-tumor	2.26±0.32	2.57±0.42	1.14±0.38	0.72±0.08	3.27±1.89	1.99±0.26	1.41±0.38

Supplemental Table 6. Biodistribution studies of [^{177}Lu]Lu-FAPI-46-Ibu in HT1080.hFAP and HEK-293.hFAP and the corresponding wild type dual xenografts at 4h, 24h, 72h and 120h p.i. Results are expressed as mean of the % injected activity per gram of tissue (%I.A./g) ± standard deviation (SD).

**Organ**	**HT-1080 xenografts**				**HEK-293 xenografts**		
	**4h (n=4)**	**24h (n=4)**	**72h (n=4)**	**120h (n=4)**	**4h (n=4)**	**24h (n=4)**	**72h (n=4)**
Blood	1.07±0.10	0.14±0.03	0.01±0.00	0.00±0.00	1.00±0.18	0.10±0.01	0.00±0.00
Heart	0.47±0.09	0.17±0.03	0.07±0.01	0.04±0.01	0.44±0.03	0.14±0.02	0.05±0.01
Lung	0.83±0.07	0.21±0.03	0.07±0.02	0.03±0.01	1.88±2.29	0.15±0.01	0.04±0.01
Liver	0.96±0.08	1.02±0.70	0.50±0.02	0.31±0.06	0.75±0.11	0.58±0.35	0.35±0.04
Pancreas	0.86±0.08	0.32±0.14	0.06±0.02	0.03±0.01	0.73±0.17	0.20±0.02	0.04±0.01
Spleen	0.45±0.13	0.28±0.07	0.21±0.04	0.11±0.02	0.32±0.05	0.24±0.02	0.13±0.07
Stomach	0.45±0.04	0.28±0.17	0.06±0.01	0.03±0.02	0.48±0.10	0.15±0.03	0.06±0.06
Intestine	0.59±0.11	0.18±0.08	0.06±0.02	0.06±0.06	0.61±0.14	0.19±0.02	0.04±0.01
Adrenal	1.38±0.14	0.33±0.04	0.19±0.03	0.12±0.03	0.85±0.19	0.24±0.15	0.13±0.03
Kidney	1.80±0.15	1.15±0.19	0.54±0.11	0.18±0.03	1.33±0.17	0.81±0.11	0.25±0.03
Muscle	0.97±0.25	0.43±0.28	0.12±0.07	0.05±0.01	0.87±0.36	0.28±0.08	0.07±0.03
Femur	3.62±0.04	1.12±0.12	0.51±0.09	0.39±0.11	2.46±0.88	0.81±0.11	0.33±0.08
hFAP-tumor	8.40±1.97	5.09±0.64	1.55±0.58	0.42±0.07	5.03±2.84	3.14±0.49	0.88±0.40
wt-tumor	1.96±0.36	1.00±0.25	0.28±0.05	0.13±0.05	1.86±1.25	0.52±0.11	0.18±0.05

Supplemental Table 7. Biodistribution studies of [^{177}Lu]Lu-FAPI-46-EB in HT1080.hFAP and HEK-293.hFAP and the corresponding wild type dual xenografts at 4h, 24h, 72h and 120h p.i. Results are expressed as mean of the % injected activity per gram of tissue (%I.A./g) ± standard deviation (SD).

**Organ**	**HT-1080 xenografts**				**HEK-293 xenografts**		
	**4h (n=4)**	**24h (n=4)**	**72h (n=4)**	**120h (n=4)**	**4h (n=4)**	**24h (n=4)**	**72h (n=4)**
Blood	11.59±0.46	5.47±0.85	1.63±0.19	0.74±0.04	13.16±2.19	6.85±0.61	1.18±0.05
Heart	3.56±0.19	2.40±0.47	1.33±0.09	1.44±0.24	4.17±0.99	2.75±0.11	1.19±0.07
Lung	5.45±0.23	3.27±0.60	1.64±0.08	1.61±0.20	6.45±1.19	3.98±0.44	1.45±0.06
Liver	2.80±0.26	2.66±0.31	2.78±0.48	2.99±0.60	3.06±0.36	3.05±0.12	2.29±0.23
Pancreas	1.71±0.15	1.41±0.50	0.69±0.05	0.74±0.13	1.77±0.22	1.45±0.21	0.60±0.06
Spleen	1.90±0.24	1.89±0.37	1.93±0.24	2.74±0.92	2.24±0.55	2.54±0.22	1.87±0.35
Stomach	1.81±0.13	1.28±0.22	0.72±0.06	0.60±0.10	2.11±0.31	1.43±0.13	0.56±0.04
Intestine	1.53±0.29	1.05±0.17	0.68±0.14	0.57±0.12	1.90±0.18	1.20±0.15	0.47±0.09
Adrenal	3.71±0.28	3.79±0.93	4.51±0.49	4.94±1.05	3.61±0.92	4.05±0.48	3.71±0.76
Kidney	4.33±0.12	5.02±0.59	5.97±0.49	7.76±2.13	4.70±1.03	6.67±0.67	4.86±0.72
Muscle	1.31±0.07	1.17±0.13	0.85±0.19	0.85±0.18	1.61±0.17	1.39±0.27	0.59±0.25
Femur	2.35±0.24	2.09±0.21	1.34±0.13	1.69±0.35	2.43±0.26	2.51±0.30	1.08±0.24
hFAP-tumor	5.02±0.29	5.44±1.03	4.16±0.19	3.95±0.89	12.58±2.83	16.69±1.01	6.75±1.06
wt-tumor	3.70±0.72	3.56±0.76	3.75±1.02	3.61±0.58	3.75±0.40	4.25±0.47	2.10±0.07

Supplemental Table 8. Biodistribution studies of [^{177}Lu]Lu-FAP-2286 in HT1080.hFAP and HEK-293.hFAP and the corresponding wild type dual xenografts at 4h, 24h, 72h and 120h p.i. Results are expressed as mean of the % injected activity per gram of tissue (%I.A./g) ± standard deviation (SD).

Organ	HT-1080 xenografts				HEK-293 xenografts		
	4h (n=4)	24h (n=4)	72h (n=4)	120h (n=4)	4h (n=4)	24h (n=4)	72h (n=4)
Blood	0.03±0.01	0.00±0.00	0.00±0.00	0.00±0.00	0.04±0.01	0.00±0.00	0.00±0.00
Heart	0.03±0.01	0.02±0.00	0.01±0.00	0.01±0.00	0.03±0.01	0.01±0.00	0.01±0.00
Lung	0.08±0.01	0.03±0.01	0.01±0.00	0.01±0.00	0.07±0.00	0.02±0.00	0.01±0.00
Liver	0.11±0.02	0.08±0.01	0.05±0.01	0.04±0.01	0.11±0.01	0.09±0.00	0.03±0.01
Pancreas	0.03±0.01	0.02±0.00	0.01±0.00	0.01±0.00	0.03±0.01	0.01±0.00	0.01±0.00
Spleen	0.05±0.00	0.03±0.01	0.02±0.00	0.02±0.00	0.04±0.00	0.03±0.00	0.01±0.00
Stomach	0.05±0.03	0.03±0.00	0.02±0.01	0.01±0.00	0.10±0.06	0.03±0.00	0.01±0.00
Intestine	0.07±0.04	0.02 ±0.01	0.01±0.01	0.01±0.00	0.09±0.07	0.02±0.01	0.01±0.01
Adrenal	0.11±0.03	0.08±0.01	0.04±0.04	0.04±0.01	0.08±0.01	0.04±0.01	0.02±0.01
Kidney	5.15±0.65	3.62±0.51	1.58±0.51	0.90±0.28	3.98±0.42	2.33±0.48	0.72±0.22
Muscle	0.04±0.01	0.02±0.01	0.01±0.00	0.01±0.00	0.06±0.03	0.02±0.01	0.01±0.01
Femur	0.15±0.05	0.10±0.01	0.16±0.06	0.06±0.01	0.27±0.04	0.20±0.01	0.04±0.02
hFAP-tumor	3.42±1.06	1.67±0.50	0.64±0.22	0.12±0.05	22.99±3.13	13.49±1.95	4.05±0.99
wt-tumor	0.10±0.01	0.08±0.01	0.04±0.01	0.02±0.00	0.28±0.13	0.13±0.05	0.02±0.02

Supplemental Table 9. Biodistribution studies of [^{177}Lu]Lu-FAPI-F1 in HT1080.hFAP and the corresponding wild type dual xenografts at 4h and 24h p.i. Results are expressed as mean of the % injected activity per gram of tissue (%I.A./g) ± standard deviation (SD).

**Organ**	**HT-1080 xenografts**	
	**4h (n=4)**	**24h (n=4)**
Blood	0.62±0.03	0.04±0.02
Heart	0.32±0.04	0.06±0.01
Lung	0.46±0.02	0.08±0.01
Liver	0.39±0.05	0.24±0.02
Pancreas	0.60±0.03	0.10±0.01
Spleen	0.27±0.03	0.17±0.03
Stomach	0.35±0.06	0.09±0.05
Intestine	0.44±0.14	0.05±0.04
Adrenal	0.83±0.08	0.13±0.07
Kidney	1.66±0.36	0.99±0.27
Muscle	0.70±0.23	0.15±0.09
Femur	3.10±0.17	0.93±0.21
HT-1080.hFAP	6.74±1.03	2.27±0.26
HT-1080.wt	1.02±0.03	0.36±0.14

***Ex vivo* blocking studies**

HT1080.hFAP and HEK293.hFAP xenografts were randomized (n=3/group), injected intravenously with the non-labeled ligand (100 µL/30 nmol of either FAPI-46 or FAP-2286) and after 5 minutes [^{177}Lu]Lu-FAPI-46 or [^{177}Lu]Lu-FAP-2286, respectively, was injected (100 µL/500 pmol/1.5 MBq). The mice were euthanized 4h after injection by CO_2 asphyxiation. Organs of interest and blood were collected, rinsed of excess blood, blotted dry, weighed, and counted in a γ-counter. The samples were counted against a suitably diluted aliquot of the injected solution as the standard and the results were expressed as the percentage of the injected activity per gram of tissue (%I.A./g) ± standard deviation (SD).

The results are presented in the **Supplemental Figures 5 and 6**.

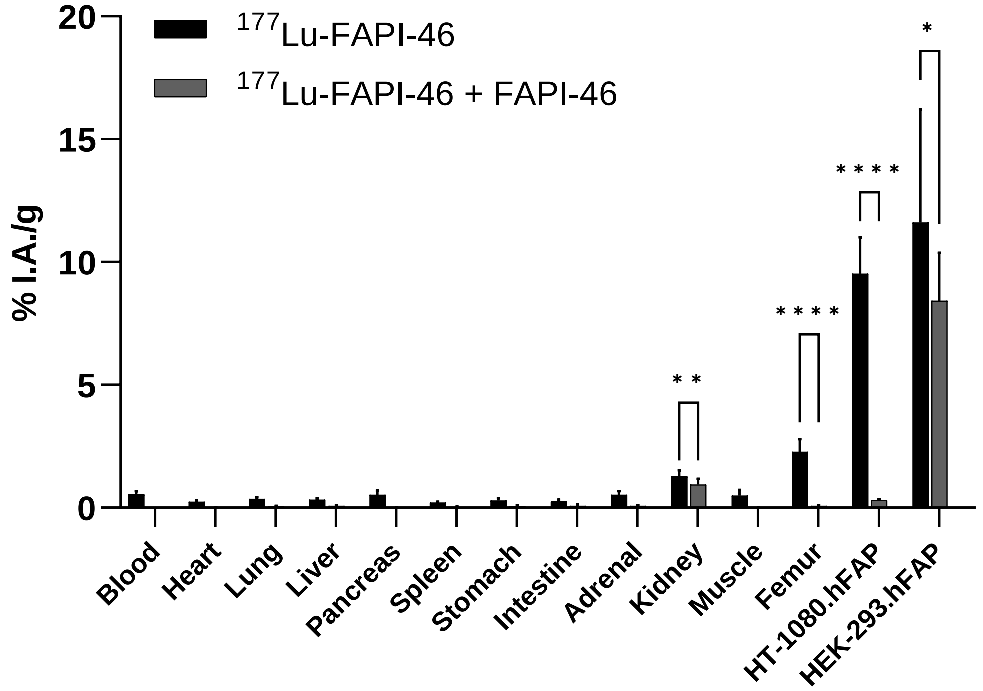

Supplemental Figure 5. Biodistribution results of [^{177}Lu]Lu-FAPI-46 in HT1080.hFAP and HEK-293.hFAP xenografts at 4h post-injection with and without 5 min pre-administration of 60-fold (30 nmol) of FAPI-46. Results are expressed as mean of the % injected activity per gram of tissue (%I.A./g) ± standard deviation (SD). **** $p<0.0001$, ** $p<0.005$ and * $p<0.05$.

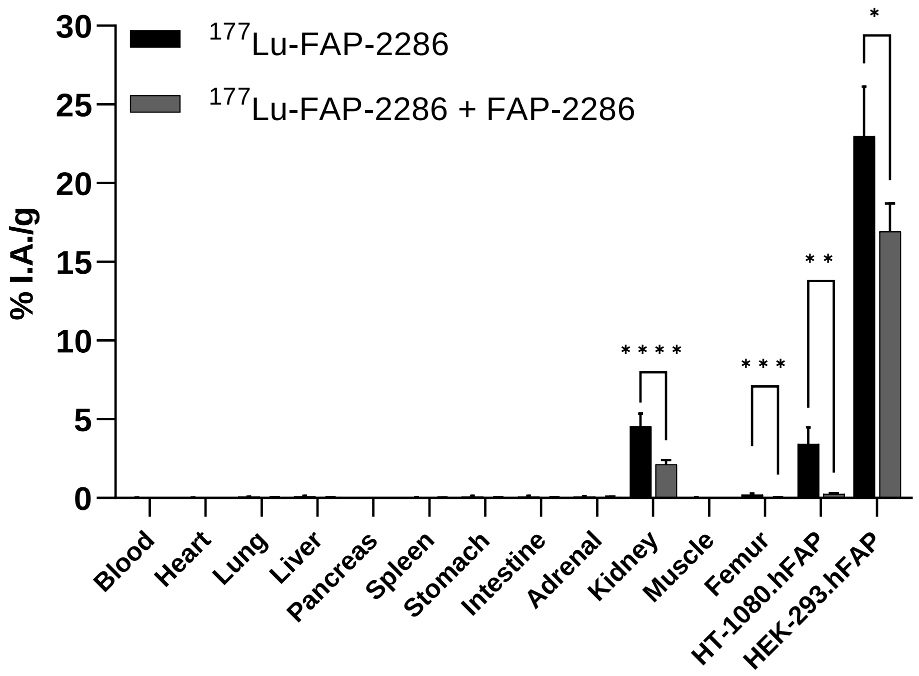

Supplemental Figure 6. Biodistribution results of [^{177}Lu]Lu-FAP-2286 in HT1080.hFAP and HEK-293.hFAP xenografts at 4h post-injection with and without 5 min pre-administration of 60-fold (30 nmol) of FAP-2286. Results are expressed as mean of the % injected activity per gram of tissue (%I.A./g) ± standard deviation (SD). **** p<0.0001, *** p<0.0005, ** p<0.005 and * p<0.05.

***In vitro* autoradiography**

HT1080.hFAP and HEK-293.hFAP tumor-bearing mice were euthanized by CO_2 asphyxiation, and the tumors were excised, snap frozen in optimal cutting temperature medium (Thermo Fisher Scientific) and stored at −80°C. The tissues were sectioned to obtain 8 µm-thick slides. The slides were incubated with [^{177}Lu]Lu-FAPI-46 or [^{177}Lu]Lu-FAP-2286 (18 MBq/nmol, 200 µL, 3 nM in PBS + 3% BSA) for 1h at room temperature, followed by 3 washing steps with PBS. Blocking studies were performed by pre-mixing the radioligand with the corresponding non-labeled ligand (200 µL, 30 µM in PBS + 3% BSA), following the same incubation and washing steps described above. Subsequently, an x-ray film (Autoradiography Cassette FBCS 810, FisherBiotech) was placed onto positioned slides and exposed for 2 days. Relative optical film density was determined using OptiQuant Version 5.0 software (PerkinElmer). Images of the slides were captured with Cytation 5 (Agilent). The results are shown in **Supplemental Figure 7**.

Higher signal for both radioligands was obtained in the high-expressing FAP HEK-293.hFAP tumor slides and lower in the low-expressing FAP HT-1080.hFAP tumor slides. In agreement with the biodistribution data, [^{177}Lu]Lu-FAP-2286 showed lower uptake than [^{177}Lu]Lu-FAPI-46 in the HT-1080.hFAP slides. Difference in their uptake in the HEK-293.hFAP slides could not be recorded due to the signal saturation.

The uptake of both radioligands was totally inhibited (complete absence of any radioactive signal in the autoradiography image) in the HT-1080.hFAP slides in the presence of 10,000-fold excess of the corresponding non-labeled ligand (blocking). In the HEK-293.hFAP slides, however, a faint signal was still recorded in the presence of the blocking, indicating lower inhibition. The findings are in agreement with the results from the *ex vivo* blocking studies.

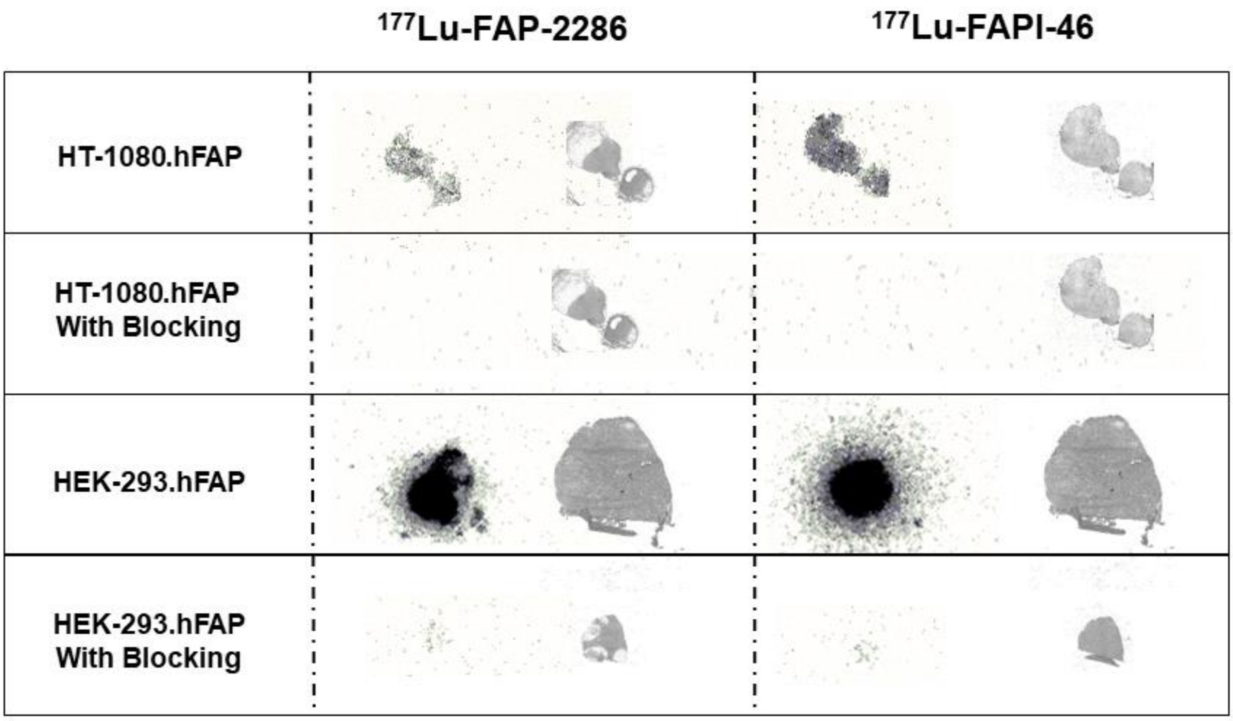

Supplemental Figure 7. *In vitro* autoradiography of [^{177}Lu]Lu-FAP-2286 and [^{177}Lu]Lu-FAPI-46 in HT-1080.hFAP and HEK-293.hFAP tumor slides without (first and third raw) or with (second and fourth raw) the presence of 10’000-fold excess of the non-labeled ligand (FAP-2286 or FAPI-46). The images of the tumor slides are provided next to each autoradiography.

Statistical analysis

Statistical analysis of the Area Under the Curve (AUC) data derived from quantitative biodistribution was performed using Prism 9 software (GraphPad Software, unpaired t-test). 95% Confidence interval (**Table 10**) and p values of AUC (mean values) are provided below.

Supplemental Table 10. 95% Confidence interval of the Area Under the Curve (AUC) of each radioligand in HT-1080.hFAP and HEK-293.hFAP tumors.

**Radioligand**	**95% Confidence interval**	
	**HT-1080.hFAP**	**HEK-293.hFAP**
**[^{177}Lu]Lu-FAPI-46**	192.4 to 294.4	112.1 to 359.0
**[^{177}Lu]Lu-FAPI-46-F1D**	357.2 to 509.4	326.4 to 554.4
**[^{177}Lu]Lu-FAPI-46-Ibu**	253.4 to 368.9	123.5 to 253.1
**[^{177}Lu]Lu-FAPI-46-EB**	291.5 to 398.4	789.1 to 971.7
**[^{177}Lu]Lu-FAP-2286**	78.60 to 148.0	705.7 to 958.0

p values of AUC

HT-1080.hFAP xenografts

[^{177}Lu]Lu-FAPI-46 vs [^{177}Lu]Lu-FAPI-46-F1D
P value 0.0002 (***)

[^{177}Lu]Lu-FAPI-46 vs [^{177}Lu]Lu-FAPI-46-Ibu
P value 0.0133 (*)

[^{177}Lu]Lu-FAPI-46 vs [^{177}Lu]Lu-FAPI-46-EB
P value 0.0016 (**)

[^{177}Lu]Lu-FAPI-46 vs [^{177}Lu]Lu-FAP-2286
P value 0.0002 (***)

[^{177}Lu]Lu-FAPI-F1D vs [^{177}Lu]Lu-FAPI-46-Ibu
P value 0.0023 (**)

[^{177}Lu]Lu-FAPI-F1D vs [^{177}Lu]Lu-FAPI-46-EB
P value 0.0097 (**)

[^{177}Lu]Lu-FAPI-F1D vs [^{177}Lu]Lu-FAP-2286
P value <0.0001 (****)

[^{177}Lu]Lu-FAPI-Ibu vs [^{177}Lu]Lu-FAPI-46-EB
P value 0.1369 (ns)

[^{177}Lu]Lu-FAPI-Ibu vs [^{177}Lu]Lu-FAP-2286
P value <0.0001 (****)

[^{177}Lu]Lu-FAPI-EB vs [^{177}Lu]Lu-FAP-2286
P value <0.0001 (****)

HEK-293.hFAP xenografts

[^{177}Lu]Lu-FAPI-46 vs [^{177}Lu]Lu-FAPI-46-F1D
P value 0.0031 (**)

[^{177}Lu]Lu-FAPI-46 vs [^{177}Lu]Lu-FAPI-46-Ibu
P value 0.2292 (ns)

[^{177}Lu]Lu-FAPI-46 vs [^{177}Lu]Lu-FAPI-46-EB
P value <0.0001 (****)

[^{177}Lu]Lu-FAPI-46 vs [^{177}Lu]Lu-FAP-2286
P value <0.0001 (****)

[^{177}Lu]Lu-FAPI-F1D vs [^{177}Lu]Lu-FAPI-46-Ibu
P value 0.0003 (***)

[^{177}Lu]Lu-FAPI-F1D vs [^{177}Lu]Lu-FAPI-46-EB
P value <0.0001 (****)

[^{177}Lu]Lu-FAPI-F1D vs [^{177}Lu]Lu-FAP-2286
P value 0.0001 (***)

[^{177}Lu]Lu-FAPI-Ibu vs [^{177}Lu]Lu-FAPI-46-EB
P value <0.0001 (****)

[^{177}Lu]Lu-FAPI-Ibu vs [^{177}Lu]Lu-FAP-2286
P value <0.0001 (****)

[^{177}Lu]Lu-FAPI-EB vs [^{177}Lu]Lu-FAP-2286
P value 0.2601 (ns)

HT-1080.hFAP vs HEK-293.hFAP

[^{177}Lu]Lu-FAPI-46
P value 0.8266 (ns)

[^{177}Lu]Lu-FAPI-46-F1D
P value 0.8458 (ns)

[^{177}Lu]Lu-FAPI-46-Ibu
P value 0.014 (**)

[^{177}Lu]Lu-FAPI-46-EB
P value <0.0001 (****)

[^{177}Lu]Lu-FAP-2286
P value <0.0001 (****)

Biodistribution in HT-1080.hFAP with 50 pmol – mass effect

To confirm that the different tumor uptake observed in the two tumor models was not due to a saturation effect in the low FAP-expressed tumors, we performed biodistribution experiments injecting 50 pmol of [^{177}Lu]Lu-FAP-2286 and [^{177}Lu]Lu-FAPI-46 at different timepoints (4h, 24h and 72h for [^{177}Lu]Lu-FAP-2286 and 4h for [^{177}Lu]Lu-FAPI-46). The results are reported below.

Supplemental Table 11. Biodistribution studies of [^{177}Lu]Lu-FAP-2286 and [^{177}Lu]Lu-FAPI-46 (50 pmol) in HT1080.hFAP and the corresponding wild type dual xenografts at the indicated times points. 4h and 24h p.i. Results are expressed as mean of the % injected activity per gram of tissue (%I.A./g) ± standard deviation (SD).

**Organ**	**[^{177}Lu]Lu-FAP-2286 (50 pmol)**			**[^{177}Lu]Lu-FAPI-46 (50 pmol)**
	**4h (n=5)**	**24h (n=3)**	**72h (n=3)**	**4h (n=3)**
Blood	0.02±0.00	0.00±0.00	0.00±0.00	1.11±0.08
Heart	0.02±0.01	0.02±0.00	0.02±0.02	0.59±0.05
Lung	0.43±0.73	0.03±0.00	0.02±0.01	0.88±0.04
Liver	0.07±0.01	0.08±0.02	0.06±0.01	0.68±0.03
Pancreas	0.03±0.01	0.02±0.00	0.02±0.02	1.55±0.09
Spleen	0.04±0.01	0.04±0.01	0.04±0.01	0.51±0.13
Stomach	0.05±0.01	0.06±0.06	0.02±0.02	0.68±0.04
Intestine	0.05±0.01	0.04 ±0.03	0.02±0.01	0.48±0.09
Adrenal	0.06±0.02	0.07±0.01	0.09±0.08	1.85±0.21
Kidney	3.32±1.21	2.42±0.40	1.21±0.25	1.38±0.04
Muscle	0.04±0.01	0.03±0.01	0.03±0.02	1.60±0.25
Femur	0.12±0.02	0.13±0.02	0.09±0.05	4.06±0.22
HT-1080.hFAP	3.44±1.68	2.00±0.37	1.51±0.67	7.76±3.17
HT-1080.wt	0.11±0.03	0.09±0.04	0.11±0.12	2.21±0.48
